# Human CD4^+^ iNKT cells have highly oxidative metabolism and use glycogen reserves to sustain IFN-γ production in nutrient-limited environments

**DOI:** 10.3389/fimmu.2026.1894113

**Published:** 2026-08-17

**Authors:** Niharika M. Patankar, Kelsey A. Smith, Nidus Jacobsen, Nikhila S. Bharadwaj, Jenny E. Gumperz

**Affiliations:** Department of Medical Microbiology and Immunology, University of Wisconsin School of Medicine and Public Health, Madison, WI, United States

**Keywords:** glucose independence, glycogen stores, interferon-gamma (IFN-g), invariant natural killer T (iNKT) cell, metabolic resilience, oxidative phosphorylation (OXPHOS), spare respiratory capacity (SRC)

## Abstract

Invariant natural killer T (iNKT) cells are promising candidates for allogeneic cellular immunotherapy, but the metabolic pathways that support their effector function in nutrient-limited tumor microenvironments remain poorly defined. Human iNKT cells segregate into CD4^+^ and CD4⁻CD8⁻ double-negative (DN) subsets with distinct functional profiles, yet whether they use divergent metabolic strategies to sustain IFN-γ production is unknown. Here we show that *in vitro*-expanded human CD4^+^ and DN iNKT cells employ distinct metabolic programs that differentially support IFN-γ secretion under nutrient stress. DN iNKT cells exhibit higher Glut1 expression and a more glycolytic phenotype, with IFN-γ production that is sensitive to extracellular glucose withdrawal and glycolytic inhibition. In contrast, CD4^+^ iNKT cells display high spare respiratory capacity, preferentially engage glutamine-supported mitochondrial respiration, and maintain IFN-γ production despite glucose deprivation or 2-DG inhibitor treatment. CD4^+^ iNKT cells generated excess ATP through oxidative metabolism, accumulated intracellular glycogen stores during expansion *in vitro*, and subsequently mobilized this glycogen to support IFN-γ production in tumor-exhausted media. Using a xenograft model of EBV-driven B cell lymphoma, we show that adoptively transferred human CD4^+^ iNKT cells infiltrate tumors *in vivo* and are associated with elevated intratumoral IFN-γ. These findings identify a distinctive combination of high mitochondrial oxidative capacity, glutamine utilization, and glycogen storage that endows human CD4^+^ iNKT cells with exceptional metabolic resilience, suggesting that CD4^+^ iNKT-based products may provide a particularly valuable platform for adoptive cellular immunotherapy.

## Introduction

Invariant natural killer T (iNKT) cells are a conserved population of innate T lymphocytes that can mediate potent anti-tumor effects [reviewed in (1–3)]. Unlike conventional T cells, which recognize peptide antigens presented by polymorphic MHC molecules, iNKT cells bear a semi-invariant T cell receptor (TCR) that recognizes lipids and glycolipids as antigens presented by CD1d, a non-classical antigen presenting molecule that has minimal polymorphism in human populations (4–6). iNKT cells are thus activated in an MHC-unrestricted manner through recognition of highly conserved ligands, a property that has made them of particular interest as a platform for allogeneic adoptive cell therapies (3, 7, 8). Like other innate lymphocytes (e.g. natural killer cells), iNKT cells produce key anti-tumor cytokines such as IFN-γ in response to endogenous activation signals (9, 10). However, little is known about the metabolic underpinnings of iNKT cell production of IFN-γ.

A central theme in immunometabolism is that lymphocyte metabolic state is an active determinant of effector function, differentiation, and survival in hostile microenvironments (11, 12). A key tenet is that the differentiation of T lymphocytes into effectors is associated with a shift from mitochondrial oxidative metabolism to aerobic glycolysis, where glucose is broken down into lactate in the cytoplasm (13). Conventional T cells thus support effector responses by upregulating aerobic glycolysis upon activation, including upregulating the glucose transporter Glut1, and increasing activity of lactate dehydrogenase A (LDHA) and glyceraldehyde 3-phosphate dehydrogenase (GAPDH) enzymes (13–15). These changes specifically facilitate IFN-γ production because Glut1 increases uptake of glucose from the extracellular milieu, the enzyme activity of LDHA is mechanistically linked to *IFNG* gene transcription (16), and the GAPDH enzyme binds to *IFNG* mRNA promoting the translation of IFN-γ protein (17). However, while a glycolytic metabolic shift promotes anti-tumor cytokine production, a high dependence on glycolytic metabolism is paradoxically associated with poor T cell anti-tumor function (18). Cancer cells rely heavily on aerobic glycolysis, commonly known as the Warburg effect, to fuel biosynthetic demands, depleting local glucose and creating a hostile environment for tumor-infiltrating lymphocytes (19, 20). Glucose competition within the TME reduces the capacity of T cells to produce IFN-γ and mediate other effector responses, and T cells dependent on glycolysis for effector function are thus at a metabolic disadvantage in this setting (21).

In contrast, despite their capacity for potent effector activity, iNKT cells appear to operate under distinct metabolic rules compared to conventional T cells. Studies in murine and human systems have shown that iNKT cells rely predominantly on oxidative phosphorylation (OXPHOS) rather than glycolysis, and that inhibition of mitochondrial OXPHOS reduces iNKT cell survival and cytokine production (22, 23). When stimulated, iNKT cells direct glucose toward the pentose phosphate pathway and OXPHOS rather than lactate production, resulting in higher net ATP generation and lower mitochondrial reactive oxygen species at steady state (24, 25). Human iNKT cells display higher expression of fatty acid oxidation and AMPK pathway genes, elevated mitochondrial mass and membrane potential, and maintain effector functions under glucose and glutamine depletion that severely impairs conventional T cell output (25). Whether iNKT cells exploit alternative fuel sources such as intracellular metabolites to sustain effector function under nutrient deprivation, remains unclear.

Additionally, it remains unclear whether distinct functional profiles of human iNKT cells are associated with differences in their metabolic strategies. Human iNKT cells can be divided into two major subsets based on CD4 co-receptor expression. CD4^+^ iNKT cells are associated with polyfunctional cytokine production encompassing both TH1 and TH2 profiles, while the CD4^-^ subset, which is mainly comprised of CD4/CD8β double negative (DN) iNKT cells, displays strong cytolytic activity and a predominantly TH1 cytokine profile (26–28). Consistent with these distinctions, DN iNKT cells mainly mediate anti-tumor effects through killing, while CD4^+^ iNKT cells have powerful adjuvant-like functions that strongly promote the anti-tumor responses of other immune effector populations (29, 30). Since iNKT cell production of IFN-γ is a key component of their anti-tumor activity, yet iNKT metabolic profiles appear to differ substantially from those of other IFN-γ producing lymphocytes, in this study we investigated the metabolic pathways responsible for IFN-γ production by human iNKT cells.

## Results

### IFN-γ production by CD4^+^ iNKT cells does not require extracellular glucose

**Figure 1:**
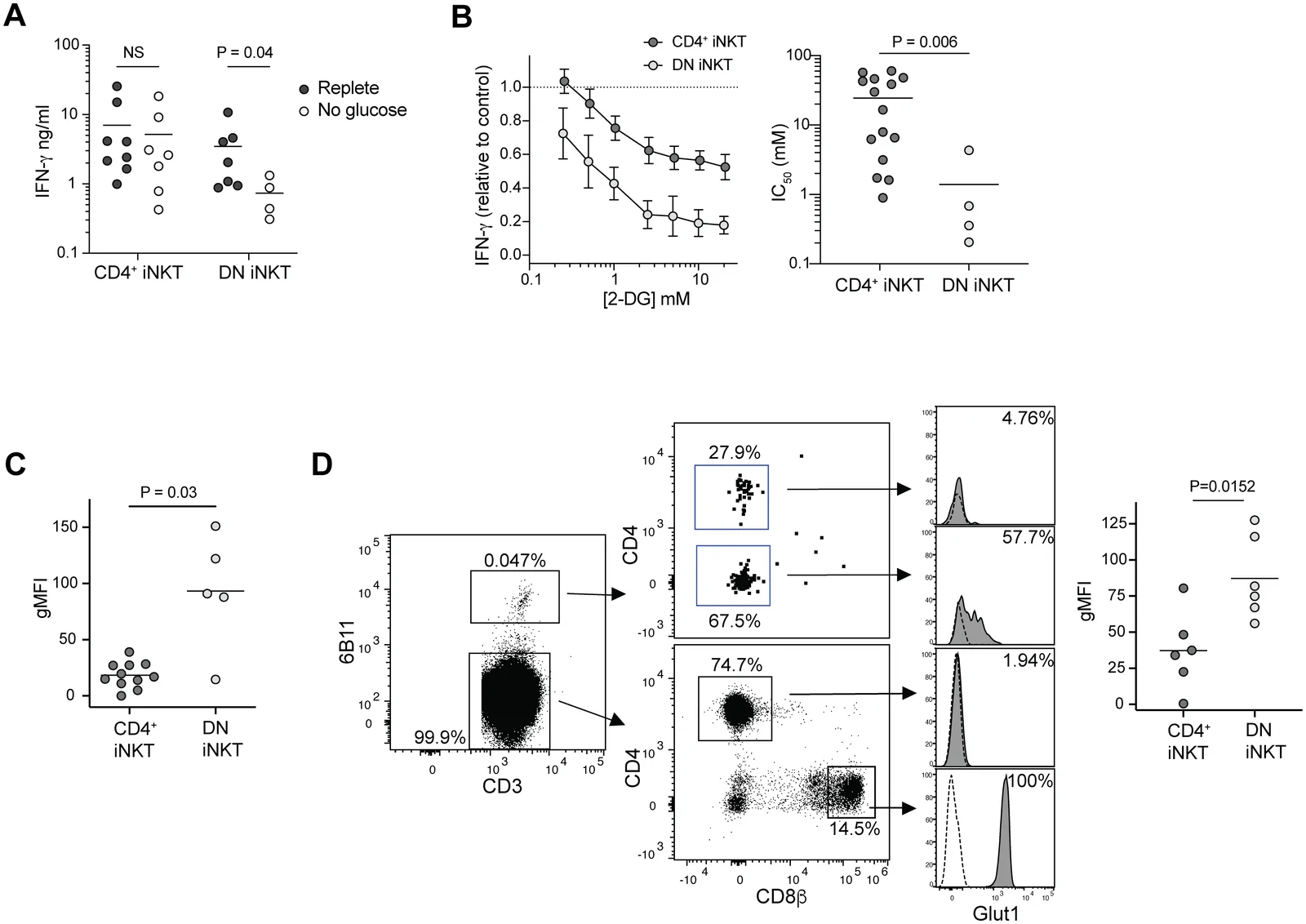
CD4^+^ iNKT cells show less reliance on glucose compared to DN iNKT cells. **(A)** CD4^+^ or DN iNKT cells were placed in nutrient-replete culture medium or in medium lacking glucose, and stimulated with an anti-CD3/anti-CD28 antibody complex for 24h. Each symbol shows mean amount of IFN-γ detected in the culture supernatant from an independent biological replicate. **(B)** iNKT cells were stimulated with anti-CD3/anti-CD28 antibody complex in nutrient-replete culture medium containing the indicated amounts of 2-DG. Plot on left shows IFN-γ (mean ± SEM) produced by CD4^+^ or DN iNKT cells in presence of 2-DG as a fraction of that produced in its absence; plot on right shows the calculated IC_50_ for each biological replicate included in the dose-response curves in the left plot. **(C)** Glut1 cell surface expression on CD4^+^ and DN iNKT cell lines was assessed by flow cytometry; symbols show geometric mean fluorescence intensity (gMFI) for each line tested. **(D)** Flow cytometric analysis of cell surface Glut1 expression by T cells in freshly isolated PBMCs. Plots on left show gating strategy and histogram overlays of Glut1 (grey filled) vs. isotype (dotted line) for a representative sample with percentages indicating the fraction of cells in the positive gate. Plot on right shows Glut1 gMFIs for CD4^+^ or DN iNKT cells from the 6 PBMC samples included in the analysis. All p values determined using two-tailed, unpaired, non-parametric t-test (Mann-Whitney).

We used human iNKT cells expanded from blood of healthy volunteer donors to assess the dependence of IFN-γ production on extracellular glucose. Resting iNKT cells were placed in glucose and glutamine-free RPMI 1640 medium supplemented with 200 mg/dL D-glucose, 2 mM L-glutamine, and 10% dialyzed bovine calf serum (BCS) (referred to here as “replete” medium), or in the same medium lacking only the glucose supplementation (glucose-free medium), and stimulated using a sub-optimal dose (10 μl/mL) anti-CD3/CD28 antibody complex. Analysis of culture supernatants after 24h revealed that CD4^+^ iNKT cells maintained nearly all of their IFN-γ production in the absence of extracellular glucose, whereas DN iNKT cells were markedly affected. The amount of IFN-γ produced by CD4^+^ iNKT cells in the absence of extracellular glucose was reduced by an average of ~25% (mean 7.0 ng/ml vs. 5.2 ng/ml), which was not a statistically significant change (**Figure 1A**). In contrast, DN iNKT cells showed an average reduction of ~80% in IFN-γ output in medium lacking glucose (mean 3.5 ng/ml vs. 0.7 ng/ml), representing a significant difference (p<0.05) (**Figure 1A**).

To further investigate, we tested the impact on iNKT cell IFN-γ production of 2-deoxyglucose (2-DG), a competitive glycolysis inhibitor. iNKT cells were placed in replete medium in the presence or absence of titrated doses of 2-DG, and stimulated with anti-CD3/CD28 antibody complex for 24h. Whereas DN iNKT cells displayed a marked dose-dependent reduction in IFN-γ with increasing concentrations of 2-DG, CD4^+^ iNKT cells showed a more modest decline and maintained ~50% of their starting cytokine production even at the highest 2-DG doses tested (**Figure 1B**). Calculated IC_50_ values for 2-DG mediated inhibition of IFN-γ secretion revealed that CD4^+^ iNKT cells required significantly higher concentrations to achieve half-maximal inhibition compared to DN iNKT cells, with mean IC_50_ values for CD4^+^ iNKT cells approximately 20-fold higher than those of DN iNKT cells (**Figure 1B**, right plot).

Consistent with this difference in glycolytic dependence of DN vs. CD4^+^ iNKT cells, we found that the two subsets showed differences in cell surface expression levels of the glucose transporter Glut1. Flow cytometric analysis of our cultured CD4^+^ iNKT cell lines consistently showed very low staining for Glut1, whereas most of our DN iNKT cell lines had substantially higher Glut1 staining (**Figure 1C**). To assess whether primary iNKT cells also show differences in Glut1 expression, we performed flow cytometric analysis of isolated peripheral blood mononuclear cells (PBMCs) using an antibody specific for the invariant TCRα chain to differentiate iNKT cells from other T cells. As expected, the non-iNKT CD8^+^ T cell subset stained brightly for Glut1, whereas the non-iNKT CD4^+^ T cell population showed low Glut1 staining (**Figure 1D**). CD4^+^ iNKT cells showed little or no Glut1 staining, whereas DN iNKT cells showed detectable Glut1 staining at significantly higher levels of expression than the CD4^+^ iNKT subset but lower than that observed for CD8β^+^ non-iNKT cells (**Figure 1D**). CD8β^+^ iNKT cells, which have previously been shown to have a highly T_H1_/cytolytic profile (31), were only sufficiently abundant for analysis in one of the six PBMC samples tested. Interestingly, analysis of this sample revealed uniformly high Glut1 staining on CD8β^+^ iNKT cells, similar to the phenotype of non-iNKT CD8β^+^ T cells (**Supplementary Figure S1**). Together these data indicate that CD4^+^ iNKT cells likely have low glucose uptake and extracellular glucose is not required for them to maintain most of their IFN-γ production, whereas DN iNKT cells likely take up higher glucose levels than the CD4^+^ subset and are more markedly affected by glucose deprivation.

### CD4^+^ iNKT cells have high oxidative capacity and preferentially use mitochondrial metabolism for IFN-γ production

**Figure 2:**
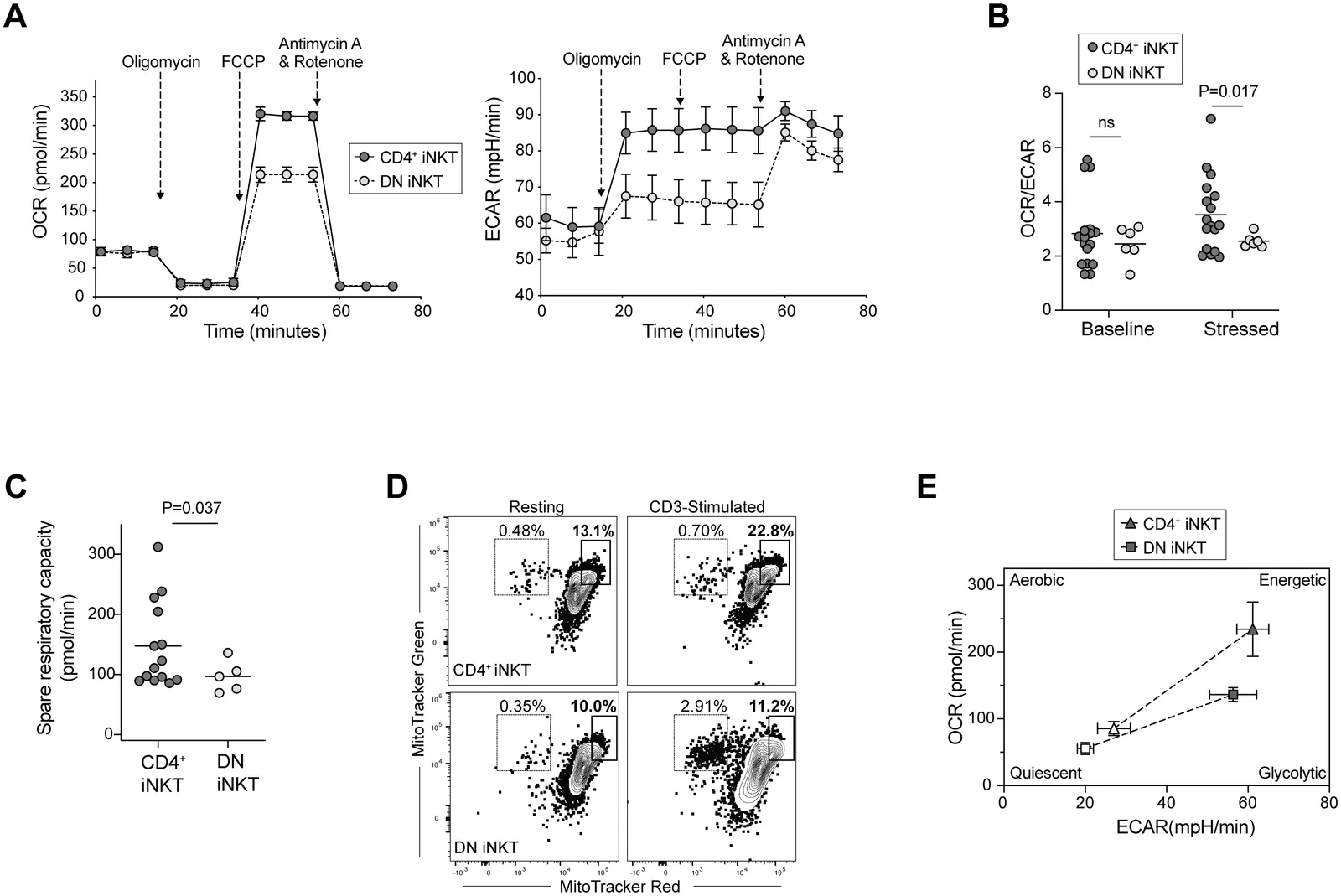
CD4^+^ iNKT cells are more preferentially biased toward mitochondrial metabolism than DN iNKT cells. Seahorse extracellular flux analysis of oxygen consumption rate (OCR) and extracellular acidification rate (ECAR) was performed on unstimulated CD4^+^ and DN iNKT cell lines. **(A)** Representative plots showing OCR (left) and ECAR (right) at baseline and after treatment with the indicated drugs that modulate mitochondrial activity. **(B)** OCR to ECAR ratio for CD4^+^ and DN iNKT cells at baseline and under stress induced by FCCP and oligomycin treatment. Each symbol shows the result from an independent biological replicate. **(C)** Spare respiratory capacity (SRC) was calculated by subtracting mean baseline OCR from maximal. Each symbol shows the result from an independent biological replicate. **(D)** Flow cytometric analysis of total mitochondrial content (MitoTracker Green) and actively respiring mitochondria (MitoTracker Red CMXRos) for two polyclonal iNKT cell lines (CD4^+^ vs. DN), before and after stimulation with anti-CD3. **(E)** Summary plot showing mean ± SEM of ECAR (x-axis) vs. OCR (y-axis) for 7 CD4^+^ (triangle symbols) and 3 DN (square symbols) iNKT cell lines. Open symbols indicate baseline, filled symbols indicate maximal. P values for **(B)** and **(C)** by unpaired t-test with Welch’s correction.

To further assess the metabolic state of our iNKT cells, we used extracellular flux analysis to evaluate mitochondrial respiration (OXPHOS) as measured by oxygen consumption rate (OCR), and cytoplasmic glycolysis as measured by extracellular acidification rate (ECAR). Unstimulated CD4^+^ and DN iNKT cells exhibited similar OCR at baseline, which was abrogated by the addition of oligomycin (an OXPHOS inhibitor), but CD4^+^ iNKT cells showed markedly higher maximal OCR levels after treatment with FCCP to drive respiration to its upper limit (**Figure 2A**, left panel). Both types of iNKT cell showed elevated ECAR after oligomycin treatment indicating increased glycolytic flux; however, ECAR upregulation was greater in the CD4^+^ line than the DN line (**Figure 2A**, right panel). Nevertheless, when OCR was normalized to ECAR, CD4^+^ iNKT cells exhibited significantly higher OCR/ECAR ratios under stressed conditions, indicating a more oxidative and less glycolytic energy phenotype than DN iNKT cells (**Figure 2B**). Calculation of spare respiratory capacity (SRC), defined as maximal minus basal OCR, indicated that the CD4^+^ iNKT cells possess significantly higher mitochondrial oxidative capacity in reserve than DN iNKT cells (**Figure 2C**). Direct assessment of mitochondrial status by flow cytometry using MitoTracker Green and Red dyes, which label total mitochondria and those that are actively respiring, respectively, revealed similar mitochondrial content and activity in unstimulated CD4^+^ and DN iNKT cells (**Figure 2D**). However, following CD3 stimulation, a larger proportion of the CD4^+^ iNKT line contained actively respiring mitochondria (high MitoTracker Red fluorescence), whereas the proportion of iNKT cells containing mitochondria with very low MitoTracker Red fluorescence, consistent with impaired mitochondrial function, was increased in DN iNKT cells (**Figure 2D**). Together these results suggest that under stress DN iNKT cell lines shift primarily toward a glycolytic phenotype, whereas CD4^+^ iNKT cells adopt an energetic phenotype, strongly increasing OXPHOS while also upregulating glycolysis (summarized in **Figure 2E**).

**Figure 3:**
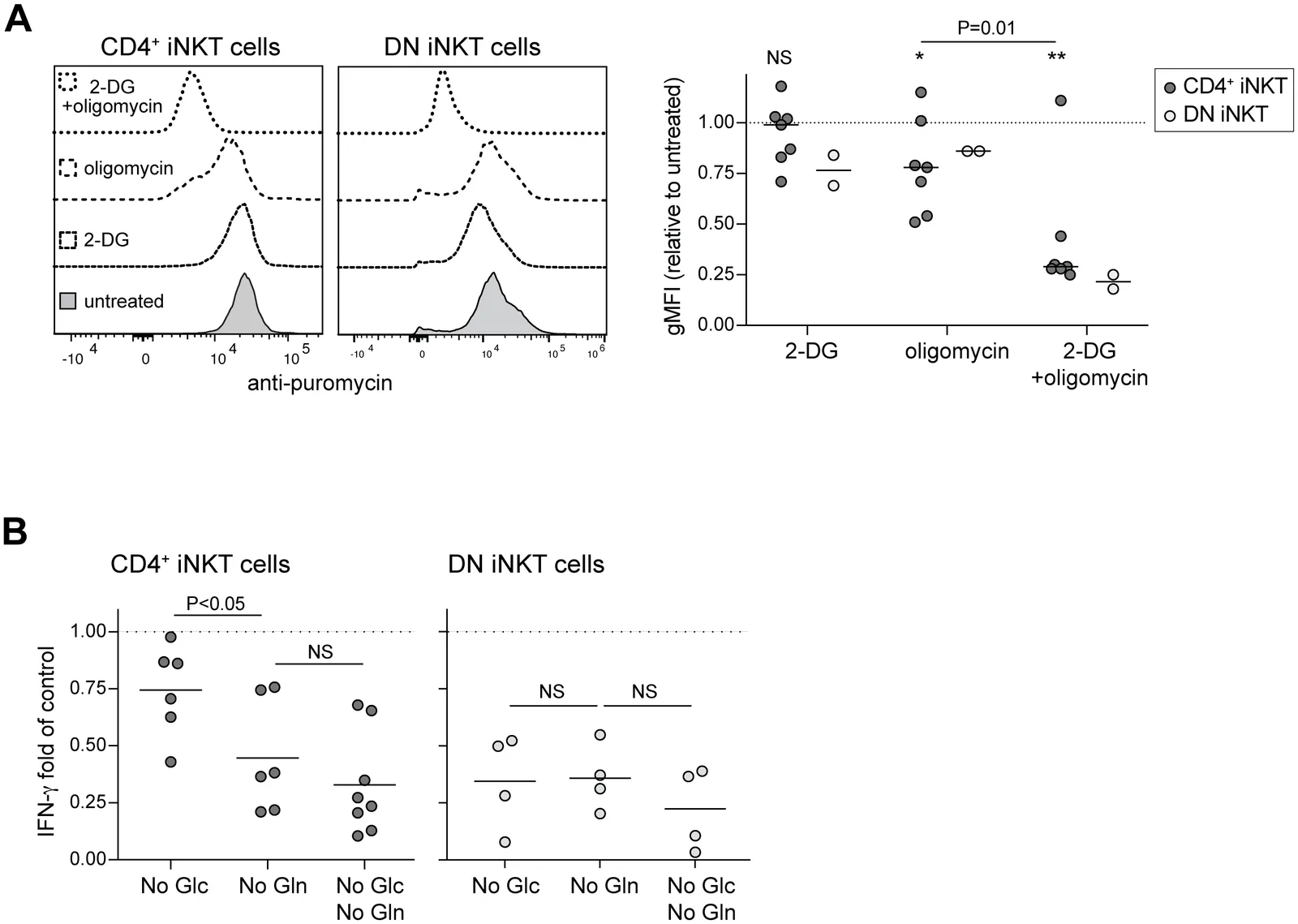
CD4^+^ iNKT cells are more dependent on mitochondrial metabolism than glycolysis for protein synthesis. **(A)** Unstimulated iNKT cells were tested for effect of blocking glycolysis via 2-DG, or mitochondrial ATP via oligomycin, on total cellular protein synthesis using puromycin incorporation as a readout. Histograms on left show representative examples of staining of CD4^+^ or DN iNKT cells cultured in nutrient-replete medium under the indicated conditions. Plot on right shows puromycin gMFI in the presence of the indicated inhibitor as a fraction of that produced in medium lacking inhibitors (“untreated”). Each symbol shows result from an independent biological replicate. Asterixes indicate significance (**p<0.01; ***p<0.001) for 1-sample t-test comparing treatment conditions to untreated control (dotted line); p value above bar shows paired t-test (two-tailed, parametric) for the indicated comparison. **(B)** CD4^+^ or DN iNKT cells were stimulated with anti-CD3/anti-CD28 antibody complex in medium lacking glucose, glutamine, or both. Each symbol shows result from an independent biological replicate. Plot shows IFN-γ levels as a fraction of that produced in parallel by iNKT cells in nutrient-replete medium (dotted line). P values by Mann-Whitney.

We next assessed the contribution of glycolysis vs. mitochondrial respiration to overall protein synthesis by CD4^+^ and DN iNKT cells. Unstimulated CD4^+^ iNKT cells were placed in replete medium in the presence or absence of 2-DG or oligomycin, then incubated with puromycin, a compound that becomes incorporated into newly synthesized polypeptides. The cells were then fixed and permeabilized, stained with an anti-puromycin antibody, and analyzed by flow cytometry to estimate the total iNKT cell protein synthesis in each culture condition as reflected by the amount of puromycin incorporation (32). Puromycin levels in CD4^+^ iNKT cells were not significantly affected by exposure to 2-DG alone, but were partially reduced (mean ~25%) in the presence of oligomycin alone and were substantially decreased (mean ~75%) by the combination of 2-DG and oligomycin (**Figure 3A**). By comparison, DN iNKT cells showed partially reduced puromycin incorporation (mean ~25%) in the presence of either 2-DG or oligomycin, while exposure to both inhibitors led to greatly reduced puromycin incorporation (mean ~80%) (**Figure 3A**). These results suggest that CD4^+^ iNKT cells rely primarily on mitochondrial respiration for steady-state cellular protein synthesis, as they maintained protein synthesis when glycolysis was inhibited, whereas inhibition of OXPHOS alone partially reduced protein synthesis. The observation that combined inhibition of both pathways resulted in a significant further reduction in protein synthesis indicates that CD4^+^ iNKT cells can shift to glycolytic ATP production when OXPHOS is compromised. In contrast, DN iNKT cells make use of both glycolysis and OXPHOS under steady-state conditions, as inhibition of either pathway alone reduces puromycin incorporation and combined inhibition nearly abolishes it.

Consistent with their reliance on both glycolysis and OXPHOS, we observed that IFN-γ production by DN iNKT cells was similarly compromised in medium lacking either glucose, glutamine, or both (**Figure 3B**, right panel), and found that in the absence of these nutrients DN iNKT cells produced only minimal absolute levels of secreted IFN-γ (**Supplementary Figure S2**). In contrast, IFN-γ production by CD4^+^ iNKT cells was only modestly affected in medium lacking glucose (**Figures 1A**, **3B**), but was significantly lower in medium lacking glutamine, which is metabolized in mitochondria (**Figure 3B**). Remarkably, there was little further decline in IFN-γ output by CD4^+^ iNKT cells in medium lacking both glucose and glutamine (**Figure 3B**), with culture supernatants in these assay conditions still often containing substantial absolute amounts of IFN-γ (mean ~3 ng/ml; **Supplementary Figure S2**). Since these results suggested that CD4^+^ iNKT cells have the capacity to maintain IFN-γ production in nutrient-deprived conditions, whereas DN iNKT cells are largely dependent on glucose and glutamine, we focused our subsequent analyses on CD4^+^ iNKT cells. As treating CD4^+^ iNKT cells with etomoxir to inhibit fatty acid metabolism did not reduce their IFN-γ production (**Supplementary Figure S3**), we turned our attention to investigating the role of intracellular glycogen stores.

### CD4^+^ iNKT cells accumulate intracellular glycogen stores that reduce their dependence on extracellular glucose

**Figure 4:**
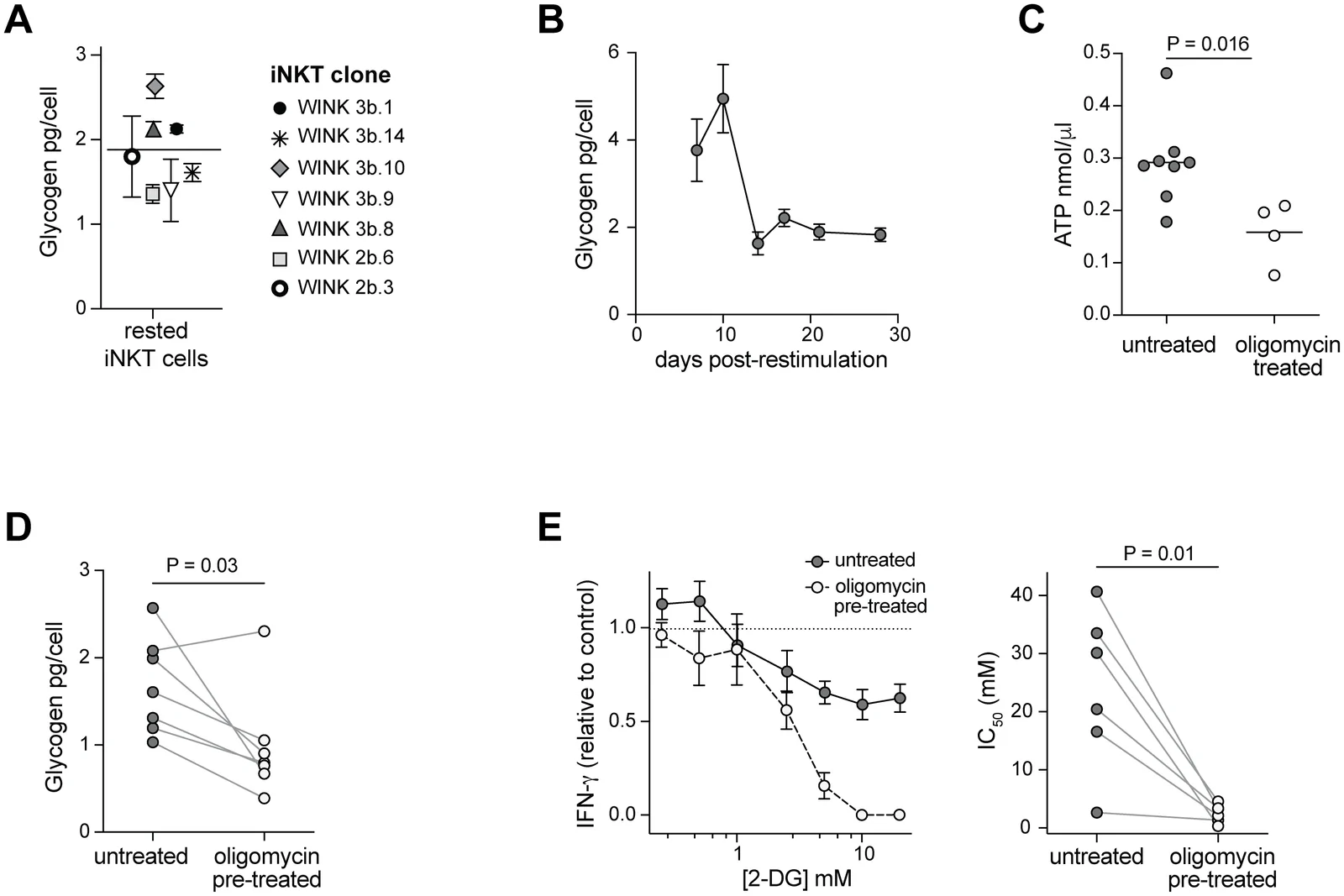
CD4^+^ iNKT cells use mitochondrially-generated ATP to produce glycogen stores. **(A)** Quantitation of intracellular glycogen levels in resting clonal CD4^+^ iNKT lines (~4 weeks post-restimulation). Symbols indicate mean ± SEM of 2–4 independent experiments for each clone. **(B)** Intracellular glycogen stores were quantified at the indicated time points following restimulation with irradiated feeder PBMCs and phytohemagglutinin (PHA). Symbols represent mean ± SEM for 6 CD4^+^ iNKT lines. **(C)** Quantification of intracellular ATP levels in CD4^+^ iNKT cells that were cultured for 48h in nutrient-replete medium alone or in the presence of oligomycin prior to analysis. Each symbol shows result from an independent biological replicate. P value by Mann-Whitney. **(D)** Analysis of intracellular glycogen levels in CD4^+^ iNKT cells that were cultured for 48h in nutrient-replete medium alone or in the presence of oligomycin prior to analysis. Each symbol shows result from an independent biological replicate. P value by paired t-test. **(E)** Six CD4^+^ iNKT cell clonal lines were either untreated or pre-treated with oligomycin to reduce glycogen stores. The iNKT cells were stimulated with anti-CD3/anti-CD28 complex in medium containing glucose but lacking glutamine in the presence of titrated doses of 2-DG to inhibit glycolysis. Plot on left shows IFN-γ levels as a fraction of that produced in parallel by the same iNKT cells in the absence of 2-DG. Symbols represent means ± SEM of six biological replicates. Plot on right shows the calculated IC_50_ for each biological replicate included in the dose-response curves in the left plot; p value determined by two-tailed paired t-test.

We quantified glycogen levels in a series of CD4^+^ iNKT cell clonal lines that were in a resting state (~ 4 weeks post-stimulation). Each iNKT line tested contained detectable glycogen, with levels ranging from 1.4-2.6 pg/cell, with a mean of 1.9 pg/cell (**Figure 4A**). Analysis of intracellular glycogen over time following restimulation to induce proliferation showed that glycogen levels peaked after about 10 days then declined by day 14 to a stable plateau that was maintained for at least two more weeks (**Figure 4B**). This pattern suggests that intracellular glycogen builds up during the period of the most rapid iNKT expansion following restimulation, but levels are maintained quite stably in culture as the cells shift to a slower rate of proliferation.

Since intracellular glycogen stores are typically generated only when cells produce excess ATP beyond their immediate cellular needs, we hypothesized that glycogen levels in CD4^+^ iNKT cells could be modulated by inhibiting mitochondrial respiration. To test this we first assessed the impact of OXPHOS inhibition on intracellular ATP levels. We found that resting CD4^+^ iNKT cells contained clearly detectable levels of intracellular ATP ranging from 0.18-0.46 nmol/μl, mean 0.29 nmol/μl (**Figure 4C**). Pre-treating them for 48h with 2 μM oligomycin led to a significant reduction in intracellular ATP levels (mean 0.16 ng/μl) without loss of viability (**Figure 4C**; **Supplementary Figure S4**). Consistent with our hypothesis, oligomycin pre-treated iNKT cells showed significantly reduced intracellular glycogen levels (**Figure 4D**).

We next performed an experiment to investigate whether stored intracellular glycogen makes CD4^+^ iNKT cells less dependent on extracellular glucose to generate IFN-γ. Rested CD4^+^ iNKT cells were pre-treated with oligomycin for 48h to reduce the levels of stored glycogen, or mock-treated. The cells were washed and then placed in medium containing glucose but lacking glutamine (to limit use of glutaminolysis), in the presence or absence of titrated doses of 2-DG to inhibit glycolysis. They were stimulated with anti-CD3/CD28 antibody complex for 24h, and IFN-γ secreted into the culture supernatant was quantitated by ELISA. IFN-γ production by oligomycin pre-treated iNKT cells was significantly more sensitive to inhibition by 2-DG, as indicated by an approximately 10-fold reduction in 2-DG IC_50_ (mean 24.0 mM to 2.5 mM) following oligomycin pre-treatment (**Figure 4E**). This suggests that iNKT cells that contained reduced stores of glycogen due to oligomycin pre-treatment were more reliant on metabolism of extracellular glucose (which is efficiently blocked by 2-DG). Together, these results suggest that CD4^+^ iNKT cells maintain intracellular glycogen stores, and can utilize this reserve to support IFN-γ production in metabolically challenging environments.

### CD4^+^ iNKT cell IFN-γ production in tumor-conditioned media is dependent on glycogen metabolism

**Figure 5:**
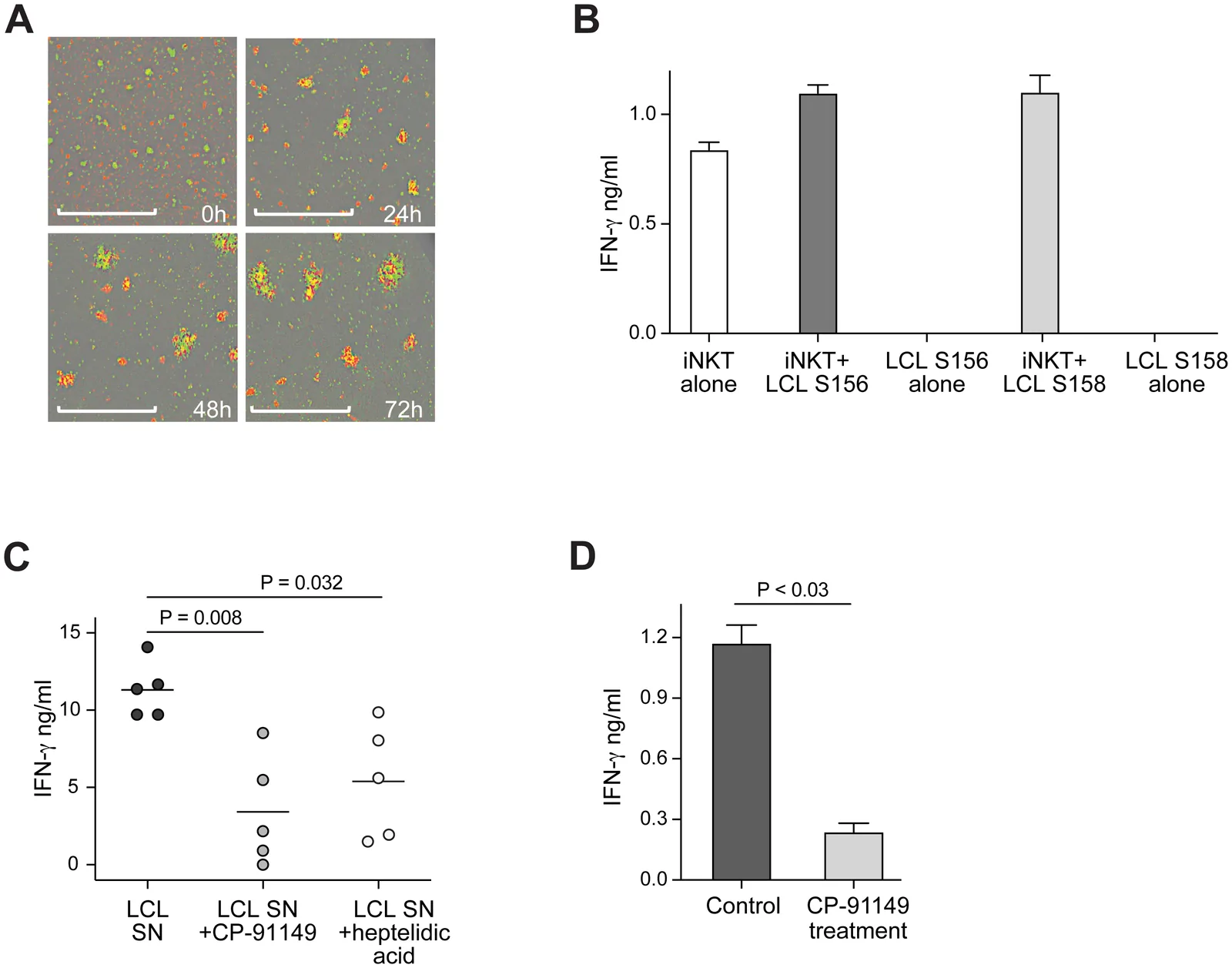
CD4^+^ iNKT cells maintain IFN-γ production in tumor-exhausted culture media through glycogen-dependent metabolism. **(A)** LCLs labeled with CFDA-SE (green) were cultured in nutrient-replete medium for 24h to allow time for spheroid formation and consumption of nutrients in medium. CD4^+^ iNKT cells labeled with CMTPX (red) were added to the culture wells and microscopic images were taken using an IncuCyte live-cell imaging platform at the indicated times. Scale bars are 2mm in length. **(B)** Quantification of IFN-γ in culture supernatants of wells containing iNKT cells alone, iNKT cells + LCLs (line S156 or S158), or the respective LCLs alone at 72h after stimulation with anti-CD3/anti-CD28 antibody complex. Bars show means ± SD of 3 technical replicates. **(C)** LCLs were cultured at high density for 48h to allow time for nutrient consumption and waste product accumulation. CD4^+^ iNKT cells were placed in filtered LCL culture supernatant alone or in the presence of the indicated metabolic inhibitors, and stimulated with anti-CD3/anti-CD28 antibody complex. IFN-γ production was quantified after 24h. Each symbol shows mean IFN-γ from an independent biological replicate; p values by Mann-Whitney. **(D)** Effect of inhibiting glycogen metabolism when glucose and glutamine metabolism are unavailable. iNKT cells were stimulated with an anti-CD3/anti-CD28 antibody complex for 24h in glutamine-free medium containing 20 mM 2-DG, in the presence of CP-91149 inhibitor or vehicle control. Bars show mean ± SEM of mean IFN-γ production from two independent biological replicate analyses.

Tumor cells have high metabolic activity that creates a significant metabolic challenge for immune cells due to nutrient depletion and build up of metabolic waste products. To investigate the ability of CD4^+^ iNKT cells to produce IFN-γ in such metabolically challenging conditions, we used Epstein-Barr virus (EBV) transformed B lymphoblastoid cell lines (LCLs) that are highly proliferative and metabolically active, and are known to consume nutrients and generate waste products quickly (33). Live-cell imaging revealed that CD4^+^ iNKT cells became closely associated with LCL spheroids within 24h of co-culture and remained associated for at least 72h (**Figure 5A**). To investigate whether this association led to impaired IFN-γ production, iNKT cells were cultured alone or with either of two independently-derived LCLs (lines S156 and S158) in the presence of a low dose of anti-CD3/CD28 antibody complex, and levels of secreted IFN-γ in the culture supernatant were quantitated after 24h by ELISA. Remarkably, IFN-γ production by CD4^+^ iNKT cells was not reduced in co-cultures with either of the two LCLs (**Figure 5B**), suggesting preservation of their effector functioning in a metabolically challenging environment.

We next investigated whether IFN-γ production by CD4^+^ iNKT cells in the context of tumor-exhausted culture medium depends on glycogen metabolism. LCLs were cultured at high density for 48-72h, and culture supernatant was collected and filtered through a 0.2 μm membrane. CD4^+^ iNKT cells were placed in this LCL medium in the presence or absence of metabolic inhibitors that inhibit glycogen breakdown, stimulated with anti-CD3/CD28 antibody complex for 24h, and secreted IFN-γ was quantitated by ELISA. We found that IFN-γ levels were significantly reduced in the presence of either CP-91149, an inhibitor of glycogen phosphorylase, or of heptelidic acid, a glyceraldehyde-3-phosphate dehydrogenase inhibitor that prevents glycogenolytic flux (**Figure 5C**). We did not observe reduced viability of iNKT cells cultured with CP-91149 or heptelidic acid, and there was no reduction in their IFN-γ production in the presence of these inhibitors in nutrient replete medium (**Supplementary Figure S5**). These results show that metabolism of intracellular glycogen plays an important role in the ability of CD4^+^ iNKT cells to produce IFN-γ in tumor-exhausted culture medium.

To further confirm this, we tested the impact of the CP-91149 inhibitor on IFN-γ production under assay conditions that prevented the iNKT cells from utilizing glucose or glutamine. CD4^+^ iNKT cells were stimulated with anti-CD3/anti-CD28 antibody complex in glutamine-free medium containing the 2-DG glycolysis inhibitor, in the presence of CP-91149 inhibitor or vehicle control. Analysis of secreted IFN-γ after 24h revealed a marked reduction (mean ~80%) in the presence of CP-91149 inhibitor compared to control (**Figure 5D**), indicating a key role for glycogen metabolism when glycolysis and glutaminolysis are unavailable.

### CD4^+^ iNKTs infiltrate tumors *in vivo* and enhance intratumoral IFN-γ

**Figure 6:**
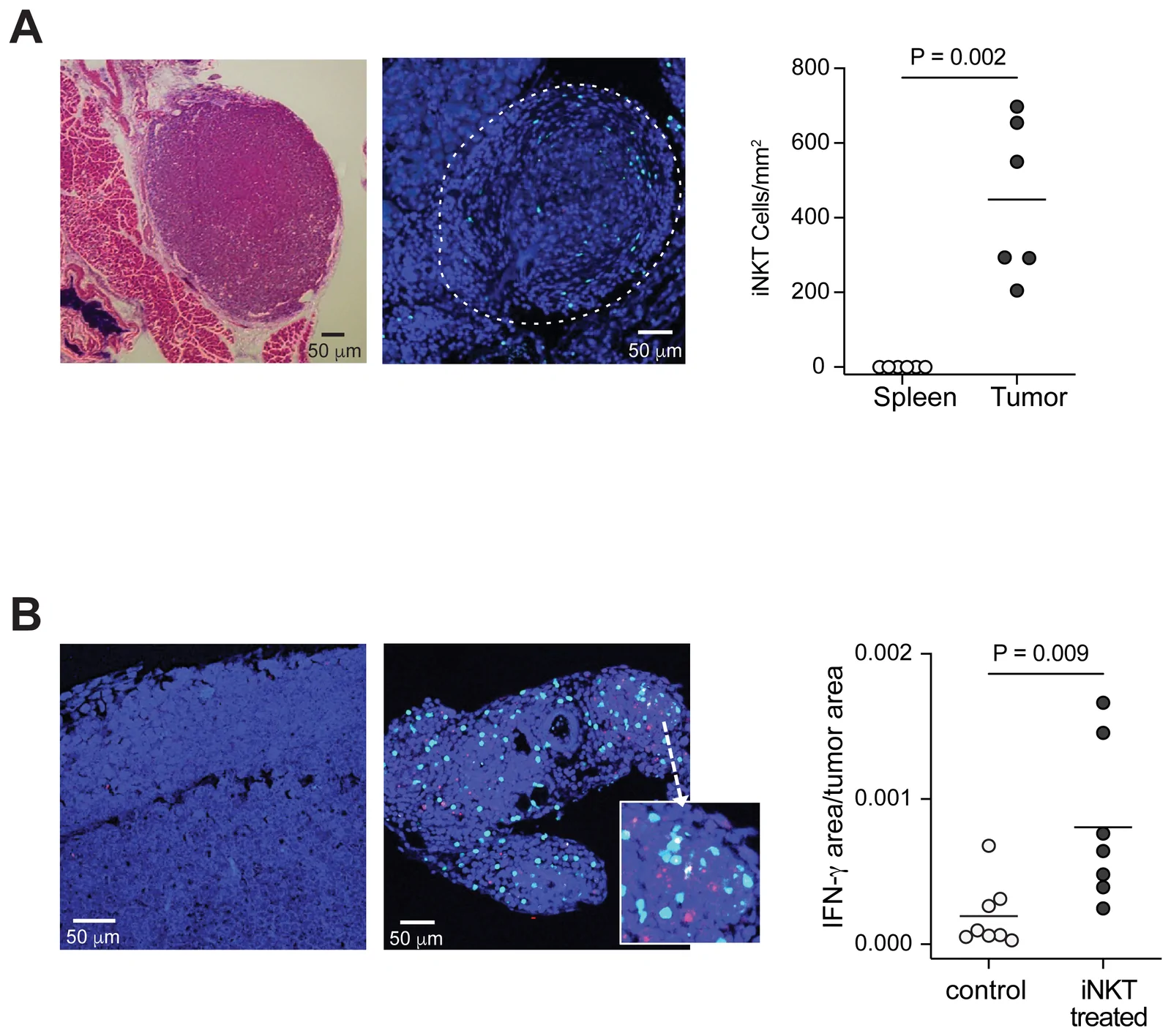
CD4^+^ iNKT cells preferentially localize to tumors *in vivo* and are associated with increased intratumoral IFN-γ. **(A)** NSG mice were intraperitoneally injected with EBV-infected human cord blood mononuclear cells (CBMCs) resulting in the formation after 3–4 weeks of EBV-driven human B cell lymphomas in the peritoneal cavity, as seen in left image showing H&E stained tumor tissue at day 26. CD4^+^ iNKT cells were labeled with CFSE (green) and injected intraperitoneally at day 25. Right image shows fluorescence microscopy analysis of tumor (outlined with white dotted line) with cell nuclei stained with DAPI (blue) and evidence of infiltrated iNKT cells (green). Plot on right shows quantitation of number of iNKT cells/mm of tumor tissue vs. spleen tissue. Each symbol represents the number of labeled iNKT cells enumerated in an independent 1 mm^2^ area of lymphoma. **(B)** Fluorescence microscopy showing IFN-γ staining (red) of tumor tissue from a non-injected control mouse (left image) or a mouse injected with CFSE-labeled iNKT cells (right image). Enlarged inset shows instances (yellow) where IFN-γ staining is directly co-localized with iNKT staining. Plot on right shows quantification of IFN-γ stained area normalized to tumor area for tumor-bearing mice given iNKT cells 24h earlier, or control mice that were not injected with iNKT cells. Each symbol represents the normalized IFN-γ staining in an independent 1 mm^2^ area of lymphoma. All p values by Mann-Whitney.

To further investigate, we utilized a previously established xenotransplant model of EBV-driven human B cell lymphoma (29, 30, 34–36), and assessed tumor infiltration by adoptively transferred human CD4^+^ iNKT cells. Lymphoma-bearing mice were generated as described previously (29, 30). Fluorescently labeled CD4^+^ iNKT cells were administered intraperitoneally, and tumors were harvested 24h later to assess iNKT cell infiltration. Immunofluorescence imaging revealed marked infiltration of adoptively transferred CD4^+^ iNKT cells into tumor tissue, with iNKT cell frequency averaging approximately 400 cells/mm^2^, but no detectable localization to spleen (**Figure 6A**). Interestingly, in similar experiments with adoptively transferred DN iNKT cells we observed significantly less localization to tumor tissue (**Supplementary Figure S6**), and instead found DN iNKT cells more abundantly localized to other sites (e.g. spleen, lymphocytic aggregates in fatty tissues). These results indicate that CD4^+^ iNKT cells are able to adapt to the metabolic constraints of the tumor microenvironment well enough to enable penetration into tumor tissue.

To assess whether they may also remain functionally active, we investigated intra-tumoral IFN-γ associated with adoptive transfer of CD4^+^ iNKT cells. Immunofluorescence analysis of tumor sections stained for IFN-γ revealed significantly elevated IFN-γ production within tumor tissue of mice that received adoptively transferred iNKT cells compared to mock-treated tumor bearing mice (**Figure 6B**). In some cases, IFN-γ staining was localized within labeled iNKT cells (**Figure 6B**), suggesting that the transferred iNKTs retain effector function within the tumor microenvironment. Collectively, these results support a cohesive model where the high spare respiratory capacity of human CD4^+^ iNKT cells is associated with increased ATP availability that can be stored as glycogen, providing intracellular stores that can subsequently be used to fuel IFN-γ production in nutrient-deprived environments such as tumor tissues.

## Discussion

In this study, we demonstrate that human CD4^+^ and DN iNKT cells adopt distinct metabolic programs that differentially support their effector functions under nutrient stress. DN iNKT cells exhibit a more glycolytic phenotype characterized by higher Glut1 expression and increased sensitivity of IFN-γ production to glycolytic inhibition and glucose withdrawal. In contrast, CD4^+^ iNKT cells show high metabolic flexibility that directly impacts their ability to produce the key anti-tumor cytokine, IFN-γ. CD4^+^ iNKT cells engage mitochondrial respiration (e.g. glutamine metabolism) to support IFN-γ production, and also generate glycogen stores that provide an intracellular metabolic resource that enables them to respond in a manner that is not dependent on extracellular glucose. CD4^+^ iNKT cells maintained nearly all of their IFN-γ production in the face of glucose deprivation, while glycolytic blockade through 2-DG limited about 50% of their response. This more pronounced effect of 2-DG relative to glucose withdrawal alone may reflect a dual mechanism of action: in addition to blocking glycolytic flux at the hexokinase/phosphoglucose isomerase step, 2-DG-6-P (a downstream metabolite of 2-DG) can act as a competitive inhibitor of glycogen phosphorylase (37), thereby also constraining the glycogenolysis-based compensatory pathway that appears to sustain IFN-γ production during glucose deprivation alone. Consistent with this glycogen-dependent resilience, CD4^+^ iNKT cells maintained strong IFN-γ production in the presence of tumor cells, but this response was highly dependent on glycogen metabolism. These findings highlight a distinctive metabolic strategy that endows CD4^+^ iNKT cells with superior functional resilience in nutrient-limited environments.

The results presented here extend and refine prior work showing that human iNKT cells, as a lineage, rely heavily on OXPHOS (22, 24, 25). Our study shows that this metabolic program is not uniform across the human iNKT compartment but is instead particularly pronounced in the CD4^+^ subset. The observation that DN iNKT cells shift predominantly toward glycolysis under metabolic stress, while CD4^+^ iNKT cells substantially increase OXPHOS in addition to glycolysis, suggests that the two subsets may be hard-wired to use different metabolic strategies to support their respective effector programs, where DN iNKT cells exhibit a cytolytic/T_H1_ phenotype, and CD4^+^ iNKT cells encompass both regulatory and pro-inflammatory capacities. Specifically, the heightened glycolytic dependence of DN iNKT cells may be well suited to rapid cytolytic responses that require burst energy and biosynthetic capacity, analogous to conventional cytotoxic T cells and NK cells whose effector functions correlate with high glycolytic flux (12, 21, 38). By contrast, the strong OXPHOS capacity and metabolic flexibility of CD4^+^ iNKT cells may favor sustained cytokine production over longer time scales in metabolically compromised environments, which is consistent with their adjuvant-like, polyfunctional role.

A surprising result from our study is that CD4^+^ iNKT cells preferentially rely on glutamine metabolism, rather than extracellular glucose, to support IFN-γ production under standard conditions. Inhibition of mitochondrial respiration with oligomycin significantly reduced total protein synthesis and IFN-γ secretion by CD4^+^ iNKT cells, while glycolytic inhibition with 2-DG alone had minimal impact on ongoing protein synthesis. Moreover, glutamine withdrawal reduced IFN-γ production more strongly than glucose deprivation, and removal of both glucose and glutamine did not significantly decrease IFN-γ output beyond glutamine withdrawal alone, suggesting that glutamine serves as the dominant fuel for mitochondrial support of effector function in this subset.

These findings contrast with the canonical model derived from conventional CD4^+^ T cells, in which effector differentiation and IFN-γ production are closely tied to aerobic glycolysis, high Glut1 expression, and the activity of glycolytic enzymes such as LDHA and GAPDH. In conventional CD4^+^ T cells, glycolysis directly supports *IFNG* transcription and translation, and a strong glycolytic shift is often necessary for robust T_H1_ effector responses in nutrient-replete conditions (16, 21). Our data suggest that CD4^+^ iNKT cells deviate from this paradigm by relying more heavily on mitochondrial oxidation of glutamine, while using glycolysis in a more auxiliary fashion that becomes important mainly when mitochondrial respiration is compromised. Consistent with this, we also noted that extracellular glucose is dispensable for steady-state protein biosynthesis by CD4+ iNKT cells (**Figure 3A**), and did not have a significant impact on their IL-4 production (**Supplementary Figure S8**). This non-canonical arrangement may provide a basis for CD4^+^ iNKT cells to sustain effector function in glucose-limited tumor microenvironments where conventional T cells are severely handicapped.

A central mechanistic insight from this work is that CD4^+^ iNKT cells accumulate substantial intracellular glycogen stores during culture and use these stores as an endogenous fuel reservoir to sustain IFN-γ production in nutrient-depleted conditions. Resting CD4^+^ iNKT cell lines 4 weeks post-stimulation contained readily detectable glycogen on the order of 1.4–2.6 pg/cell, and kinetic analysis after restimulation showed a peak around day 10 followed by a stable plateau through at least day 28. This trajectory suggests that glycogen is built up during periods of rapid expansion and then maintained as a long-term metabolic reserve as proliferation slows. Importantly, depleting glycogen stores via oligomycin pretreatment greatly increased the sensitivity of their IFN-γ production to 2-DG, suggesting that glycogen availability is key to their insensitivity to glycolytic blockade. These data support a model in which mitochondrial respiration enables CD4^+^ iNKT cells to convert excess ATP into stored glycogen, which can later be mobilized to fuel IFN-γ production when extracellular glucose is scarce or glycolysis is constrained.

These findings extend growing evidence that glycogen supports diverse aspects of T cell function. In memory CD8^+^ T cells, PCK1 serves as a metabolic hub connecting glycolysis, TCA-cycle carbon, and gluconeogenesis to glycogen synthesis, with subsequent glycogenolysis supporting pentose phosphate pathway-dependent redox homeostasis and memory T cell fitness (39). During recall, TCR signaling directly activates PYGB-dependent glycogenolysis to provide the initial glucose-6-phosphate required for rapid responses (40), while human MAIT cells similarly use stored glycogen to support rapid cytotoxicity and cytokine production (41). More recently, glycogen-derived glucose-1-phosphate was shown to promote G6PD oligomerization and compartmentalization of pentose phosphate pathway enzymes in memory CD8^+^ T cells, suggesting that glycogen can support T cell responses through both energy provision and redox regulation (42). Our results extend these observations to human CD4^+^ iNKT cells by showing that glycogen stores can support IFN-γ production during nutrient stress. Importantly, because our iNKT cells were expanded and maintained in medium containing both glucose (8.9 mM) and serum, it is likely that serum-derived insulin and/or the glucose present in the culture medium may have impacted glycogen synthesis. Thus, a key area for further investigation will be to understand how culture conditions impact glycogen stores in immunotherapeutic iNKT cells.

Our analysis suggests the metabolic strategy of CD4^+^ iNKT cells may be highly relevant for their anti-tumor activity. We found that CD4^+^ iNKT cells maintained robust IFN-γ production when co-cultured with highly metabolically active LCL spheroids, despite the expected nutrient competition and accumulation of waste products. Complementing this, we also showed in our murine xenotransplant model of EBV-driven B cell lymphoma that adoptively transferred human CD4^+^ iNKT cells infiltrated tumor tissues and were associated with significantly elevated IFN-γ production there. Importantly, IFN-γ levels were significantly reduced when the CD4^+^ iNKT cells were stimulated in LCL-conditioned medium in the presence of inhibitors that specifically block glycogen use, indicating that glycogen metabolism is critical for sustaining their cytokine production in metabolically hostile conditions. These findings build on our prior work showing that CD4^+^ iNKT cells promote clearance of EBV-driven lymphomas through adjuvant-like mechanisms, including the recruitment and activation of other immune effectors within the tumor microenvironment (29, 30). Specifically, the ability to draw upon glycogen stores provides a critical metabolic basis for sustained IFN-γ production that may contribute to tumor microenvironment remodeling, including enhanced MHC and co-stimulatory molecule expression on antigen-presenting cells, altered chemokine profiles, and facilitation of conventional T and NK cell recruitment and activation.

Several limitations of this study warrant consideration. First, these experiments were performed using clonal and polyclonal iNKT cell lines isolated from peripheral blood of healthy donors, that were subjected to multiple rounds of restimulation *in vitro*. While we believe that these iNKT cells are similar to those produced for use as allogeneic immunotherapies, it is possible that iNKT cells expanded under clinical-grade conditions or using other expansion protocols may differ. Moreover, as we used resting iNKT cells for our metabolic analyses, it will be critical in future studies to rigorously assess metabolic phenotypes of TCR- or cytokine-activated iNKT cells. Similarly, although we confirmed key differences in Glut1 expression on primary peripheral blood iNKT subsets, further work is needed to determine the stability of the glycogen-producing metabolic phenotype of CD4^+^ iNKT cells across donors and under distinct expansion protocols. Second, the experimental systems used here may not fully recapitulate the complexity of human tumor microenvironments, which involve factors such as hypoxia, spatial gradients, and stromal cell populations that modify immune responses. Addressing these limitations will require further validation in other model systems and through additional experimental strategies.

Collectively, our findings provide evidence that CD4^+^ iNKT cells possess a distinctive combination of high spare respiratory capacity, glutamine-supported mitochondrial metabolism, and glycogen storage that together confer marked metabolic resilience and sustained IFN-γ production in nutrient-deprived conditions. This profile is particularly attractive in light of the field’s shift away from repeated *in vivo* activation of endogenous iNKT cells with lipid antigens—an approach limited by reduced iNKT frequency and hyporesponsiveness in many cancer patients (43, 44), and by concerns about exhaustion or skewing toward regulatory phenotypes with repeated glycolipid dosing (45, 46)—and toward adoptive iNKT cell immunotherapies expanded *ex vivo* (2, 8, 47–51). Recent early-phase clinical trials have shown that allogeneic iNKT cell therapies can be administered safely and can mediate anti-tumor activity [reviewed in (3)], underscoring the need to understand and exploit their metabolic properties. Our data suggest that CD4^+^ iNKT cells, by virtue of their robust mitochondrial respiration and capacity to build and mobilize glycogen stores, may provide a highly metabolically resilient platform for adoptive cell therapy, particularly in solid tumors where glucose competition and generalized nutrient stress are major barriers to T cell persistence and function. Future work should explore how manufacturing conditions, such as cytokine combinations, nutrient composition, and pharmacologic or genetic modulation of metabolic pathways, can be optimized to further enhance mitochondrial fitness and glycogen reserves in therapeutic iNKT products. It will also be important to determine how these metabolic features interface with engineered receptors or co-stimulatory domains in CAR-iNKT cells, and whether similar glycogen-dependent mechanisms contribute to resistance against other suppressive features of the tumor microenvironment, such as hypoxia, high lactate, or immunosuppressive metabolites. Overall, this study advances our understanding of how human CD4^+^ iNKT cells sustain effector function under metabolic stress and offers a conceptual and mechanistic foundation for developing iNKT-based therapies that are tailored to thrive in hostile metabolic landscapes.

## Materials and methods

### Bioethics

#### Human blood samples

All use of human tissue samples was performed in accordance with the U.S. Common Rule and with Institutional Review Board (IRB) approval. Peripheral blood samples were collected under written informed consent from 12 healthy, non-pregnant adult donors (age 18-65) in accordance with University of Wisconsin IRB protocol #2018-0304. Five de-identified human umbilical cord blood samples obtained from accredited suppliers were used in accordance with University of Wisconsin IRB exemption #2023-0392.

#### Animal studies

A total of 20 mice were used for this study. Animal husbandry and all experimental procedures were approved by the University of Wisconsin IACUC, under protocol #M005199.

### iNKT cells

Polyclonal and clonal CD4^+^ and DN iNKT cell lines were generated from PBMCs of healthy adult donors as previously described (29). Briefly, CD4^+^ or CD4^-^CD8β^-^ iNKT cells were sorted using human CD1d tetramers loaded with the PBS-57 lipid analogue of α-GalCer (NIH tetramer facility, Emory University), and expanded in the presence of irradiated PBMCs and 1 μg/ml phytohemagglutinin (Sigma-Aldrich Cat# L1668), in “T cell culture medium” [RPMI1640 containing 2mM L-glutamine, 10% heat-inactivated fetal bovine serum (FBS) (GeminiBio Cat# 100-106), 5% heat-inactivated bovine calf serum (BCS) (HyClone Cat# SH30072.03), 3% pooled human AB serum (GeminiBio Cat# 100-812) supplemented with 200U/ml recombinant human IL-2 (Peprotech Cat# 200-02). iNKT cells were then maintained in T cell culture medium supplemented with IL-2, and re-stimulated by exposure to irradiated PBMCs and PHA as needed. iNKT cell cultures were regularly tested using PBS-57 loaded CD1d tetramer or 6B11 mAb (BioLegend Cat# 342916 RRID: AB_2721323) to confirm that purity was >98%, and that the cultures were either CD4^+^ or CD4^-^CD8β^-^ (DN), as indicated. **Supplementary Table 1** provides specific information on iNKT cell lines used in this study.

### Analysis of iNKT cell IFN-γ production in nutrient-defined culture media

Experiments were carried out using culture medium made using RPMI 1640 medium lacking both glucose and glutamine (Biological Industries Cat# 01-101-1A), to which 200 mg/dL D-glucose or 2 mM L-glutamine were added as indicated; all media were supplemented with 10% BCS that was first heat-inactivated and dialyzed using a 10,000 MWCO membrane. iNKT cells were placed in 200 μl of the indicated culture medium at 5 × 10^4^ cells per well in 96-well plates and stimulated with 10 μl/mL anti-CD3/CD28 antibody complex (ImmunoCult™, Stemcell Technologies) and incubated in a humidified incubator at 37°C and 5% CO_2_. Culture supernatants were harvested after 24h, and IFN-γ was assessed by ELISA (Biolegend) using 3–6 technical replicate wells and quantified by comparison to 3 technical replicate wells containing recombinant human IFN-γ standards (Biolegend). Glycolytic inhibition studies were performed using media containing 200 mg/dL D-glucose and 2 mM L-glutamine in the presence of the indicated concentrations of 2-deoxy-D-glucose (2-DG) (Thermo Fisher Scientific Cat# 111980010). IC_50_ values for 2-DG-mediated inhibition were calculated using non-linear regression (GraphPad Prism). CD4^+^ iNKT cells used for these assays included a total of 10 clonal and 1 polyclonal line, representing 3 unrelated donors; DN iNKT cells included 2 polyclonal and 1 clonal line, representing 2 unrelated donors.

### Flow cytometric analysis of Glut1 expression

Cultured iNKT cells or freshly isolated total PBMCs were resuspended in phosphate buffered saline (PBS) containing 5% BCS, 20% human AB serum, and 0.1% sodium azide (“FACS buffer”), and stained for 20 min at 4 °C using the following fluorescently labeled antibodies: AlexaFluor700-labeled anti-CD3 (Clone HIT3a, Biolegend Cat # 300324), BrilliantViolet421-labeled anti-CD4 (Clone A161A1, Biolegend Cat # 357424), PECy7-lableled anti-CD8β (Clone SIDI8BEE; Invitrogen Cat # 25-5273-42, PE-labeled anti-Vα24-Jα18 TCR (clone 6B11, Biolegend Cat # 342904), and AlexaFluor488-labeled anti-Glut1 (Clone 202915, R&D Systems Cat # FAB1418G), or isotype for Glut1 AlexaFluor488-labeled IgG2b (Clone RMG2b-1, Biolegend Cat # 406718). The cells were washed and resuspended in FACS buffer, and analyzed using an AttuneNxT flow cytometer (Thermo Fisher Scientific). Single color control compensation tubes were run for each fluorophores using positive control (Cat # 51-90-9001229) and negative control (51-90-9001291) compensation beads from BD Biosciences. Data were analyzed using FlowJo v10 software (BD Biosciences). **Supplementary Figure 7** shows upstream gating for the analyses shown in **Figure 1D**.

### Extracellular flux analysis of metabolic activity

Analyses of oxygen consumption rate (OCR) and extracellular acidification rate (ECAR) were performed using a Seahorse XF Analyzer (Agilent Technologies). Mitochondrial activity was evaluated using the Seahorse Mito Stress Test (Agilent Technologies) following the manufacturer’s protocol. Cells were seeded at a density of 2.5 × 10^5^ cells/well in 4–5 replicate wells in culture plates that were pre-coated with 50 μg/ml poly-L-lysine (Advanced biomatrix). Following baseline OCR measurements, wells were treated with 1-2 μM oligomycin, 1 μM carbonyl cyanide p-trifluoromethoxy-phenylhydrazone (FCCP) and 0.5 μM Rotenone/Antimycin A mixture (all from Agilent Technologies) at the indicated timepoints. OCR measurements from the respective assay conditions were used to determine basal respiration and maximal respiration, and these values were used to calculate spare respiratory capacity. The overall cellular metabolic phenotype of CD4^+^ and DN iNKT cells was assessed using the Seahorse Cell Energy Phenotype Test (Agilent Technologies). After setting up plates as described above and acquiring baseline OCR and ECAR measurements, assay wells were co-injected with 1 μM oligomycin and 1 μM FCCP to allow analysis of OCR and ECAR under conditions of maximal metabolic stress. CD4^+^ iNKT cells used for these assays included a total of 7 clonal and 5 polyclonal lines, representing 5 unrelated donors; DN iNKT cells included 2 polyclonal and 1 clonal line, representing 2 unrelated donors.

### Flow cytometric analysis of mitochondria

iNKT cells were incubated in pre-warmed culture medium containing 50 nM MitoTracker Red CMXRos dye (ThermoFisher Cat# M7512) and 100 nM MitoTracker Green dye (ThermoFisher Cat# M7514) for 30 min at 37 °C. Cells were washed and resuspended in fresh warm culture medium and read immediately by flow cytometry. Single stained iNKT cells were used for compensation. Data were analyzed using FlowJo v10 software (BD Biosciences), with upstream gating performed on the basis of light scatter (FSC-H vs FSC-A to include singlets and exclude debris, and FSC vs SSC to identify live cells).

### Analysis of total cellular protein synthesis

Protein synthesis was assessed using a puromycin incorporation assay as described previously (32). Briefly, iNKT cells were incubated in nutrient-replete culture medium alone or containing 2 μM oligomycin or 10 mM 2-DG or both for 15 minutes in a humidified incubator at 37 °C and 5% CO_2_. The cultures were then supplied with 10 μg/ml puromycin and incubated for an additional 30 min. Cells were fixed and permeabilized and stained using an anti-puromycin antibody (Biolegend, Clone 2A4) to detect puromycin incorporation into recently synthesized proteins. Samples were analyzed using an AttuneNxT flow cytometer (Thermo Fisher Scientific), and staining data were processed using FlowJo v10 software (BD Biosciences). CD4^+^ iNKT cells used for these assays included a total of 2 clonal and 4 polyclonal lines, representing 4 unrelated donors; DN iNKT cells included 1 polyclonal and 1 clonal line, representing 2 unrelated donors.

### Quantification of intracellular glycogen and ATP

Intracellular glycogen was quantified using a colorimetric assay (Abcam Cat# AB282931), in which cells are lysed and glucoamylase acts on glycogen, and the resulting glucose is oxidized to form an intermediate that generates a colored product with developer solutions. Each samples was tested using 5 technical replicate wells and quantitated by comparison to glycogen standards run in parallel. Intracellular ATP was quantified using a colorimetric assay (Abcam Cat # AB83355), in which cells are lysed and enzymes are used to generate a colored product in a manner that is proportional to ATP availability; ATP levels in cell lysates (2 technical replicates per sample) were quantitated by comparison to ATP standards run in parallel. Glycogen analysis was performed on 9 clonal CD4^+^ iNKT lines, and ATP analysis was performed on 8 clonal CD4^+^ iNKT lines.

### iNKT cell IFN-γ production in presence of LCL spheroids or conditioned medium

The EBV-transformed lymphoblastoid cell lines (LCLs) S156 and S158 were generated from B cells isolated from two genetically unrelated human spleen samples as described previously (29), and were maintained at 37 °C and 5% CO_2_ in RPMI 1640 medium supplemented with 10% heat-inactivated BCS, 1% penicillin/streptomycin, and 1% L-glutamine. For live-cell imaging analysis LCLs were labeled for 10 minutes at 37 °C with 5 μM CFDA-SE (ThermoFisher Cat# V12883), then washed and cultured in flat-bottom 96-well plates (Corning Cat# 3799) for 24 hours to allow spheroid formation. CD4^+^ iNKT cells were labeled with 5 μM CMTPX (Life Technologies Cat# C34552), then washed and added at a 3:1 ratio to the LCLs. Imaging of the co-cultures was performed on 5 technical replicate wells per condition. The wells were imaged in a humidified incubator at 37°C and 5% CO_2_, using an IncuCyte S3 Live-cell Imaging and Analysis System (Sartorius) with a 4x objective. Cell clustering and co-localization were analyzed using the IncuCyte platform whole well analysis software.

For assessment of iNKT cell IFN-γ production in LCL co-cultures, 10 μl/mL anti-CD3/CD28 antibody complex (ImmunoCult™, Stemcell Technologies) was added to the wells at the beginning of the co-culture. For analysis of iNKT cell IFN-γ production in LCL-conditioned media, LCLs were grown at high density for 48–72 hours then culture supernatant was collected and 0.2 μm filtered and immediately used for experiments. CD4^+^ iNKT cells (5 × 10^4^ per well) were resuspended in LCL-conditioned medium containing 10 μl/mL anti-CD3/CD28 antibody complex in the presence or absence of the glycogen phosphorylase inhibitor CP-91149 (100 μM) or the glyceraldehyde-3-phosphate dehydrogenase inhibitor heptelidic acid (5 μM), and cultured for 24h in a humidified incubator at 37°C and 5% CO_2_. Culture supernatants were analyzed for IFN-γ by ELISA using 3–5 technical replicates per condition.

### *In vivo* lymphoma model

We used a xenotransplant model of EBV-driven human B cell lymphoma that we have described in detail previously (29, 30, 34–36). Immunodeficient NOD/SCID/γ_c_^-/-^ (NSG) mice (Jackson Laboratories) were maintained in a specific pathogen free facility with aseptic housing that used sterilized bedding, food and water. Mice were 6–12 weeks of age when used. Only male mice were used for analysis due to lack of availability of female mice at the time of the study; however, we have previously shown that there is no difference in this model between male and female mice in regards to tumor burden or other outcomes (29, 30). Primary human cord blood mononuclear cells (CBMCs) were treated for 2h at 37 °C with 2,000 U of the M81 strain of EBV prepared as described previously (29, 30). Mice were anesthetized by isoflurane inhalation (3–5% isoflurane delivered in an induction chamber) and EBV-exposed CBMCs were injected intraperitoneally at 1×10^7^ cells per mouse. After 25–31 days (a period in which tumors are reliably present but not yet lethal), mice were anesthetized as above and intraperitoneally injected with 1×10^7^ CFSE-labeled CD4^+^ iNKT cells (4 mice) or DN iNKT cells (8 mice). Mice were euthanized by CO_2_ inhalation at 24h post-transfer of iNKT cells. For euthanasia, 100% CO_2_ was introduced into the chamber at a flow rate corresponding to 30–70% of the chamber volume per minute. Following exposure to CO_2_, death was confirmed by the absence of respiration (respiratory arrest), and spleen and peritoneal organs containing macroscopically visible tumor tissue were harvested and fixed for analysis.

### Immunofluorescence microscopy

Fixed tumor tissues and spleen were embedded in paraffin, and sectioned. Sections were deparaffinized, rehydrated, and subjected to heat-mediated antigen retrieval. Tissue sections were stained overnight at 4 °C with anti-human IFN-γ antibody (Biocare Cat #ACI3266A) at 1:100 dilution, then washed and incubated with a fluorescently labeled secondary antibody (Goat anti-Rabbit IgG-AlexaFluor 594, Molecular Probes, Cat # A11010) for 1 hour at room temperature, then counterstained with 1 μg/mL DAPI (Invitrogen Cat# D357). Coverslips were mounted under ProLong Gold (Invitrogen Cat# P36970) and images were acquired with a confocal microscope using a 20x objective on a Zeiss LSM 800 confocal microscope, using Zen 3.4 software. CFSE-labeled iNKT cells were quantified by counting fluorescent cells per tissue section and density was calculated as cells per mm^2^ tumor area. IFN-γ positive area was quantified using threshold-based segmentation in Fiji (ImageJ) software and expressed as a percentage of total tumor area.

### Statistical analysis

Data are presented as mean ± SEM unless otherwise indicated. Data distribution was assessed for normality and parametric or non-parametric statistical tests were selected based on the distribution of the data. Statistical tests used for analysis are indicated in figure legends. For datasets containing unpaired samples, a two-tailed, non-parametric t-test (Mann-Whitney) was typically used. Matched samples were analyzed using a two-tailed paired t-test (parametric) or Wilcoxon test (non-parametric). P values < 0.05 were considered statistically significant. All statistical analyses were performed using GraphPad Prism software.

## Data Availability

The raw data supporting the conclusions of this article will be made available by the authors, without undue reservation.

## References

[1] Wolf BJ Choi JE Exley MA . Novel approaches to exploiting invariant NKT cells in cancer immunotherapy. Front Immunol. (2018) 9:384. doi: 10.3389/fimmu.2018.00384 29559971 PMC5845557

[2] Nelson A Lukacs JD Johnston B . The current landscape of NKT cell immunotherapy and the hills ahead. Cancers (Basel). (2021) 13. doi: 10.3390/cancers13205174 34680322 PMC8533824

[3] Takami M Motohashi S . Comparative assessment of autologous and allogeneic iNKT cell transfer in iNKT cell-based immunotherapy. Front Immunol. (2024) 15:1457771. doi: 10.3389/fimmu.2024.1457771 39224603 PMC11366658

[4] Dellabona P Padovan E Casorati G Brockhaus M Lanzavecchia A . An invariant V alpha 24-J alpha Q/V beta 11 T cell receptor is expressed in all individuals by clonally expanded CD4-8- T cells. J Exp Med. (1994) 180:1171–6. doi: 10.1084/jem.180.3.1171 8064234 PMC2191638

[5] Exley M Garcia J Wilson SB Spada F Gerdes D Tahir SM . CD1d structure and regulation on human thymocytes, peripheral blood T cells, B cells and monocytes. Immunology. (2000) 100:37–47. doi: 10.1046/j.1365-2567.2000.00001.x 10809957 PMC2326993

[6] Gumperz JE . Antigen specificity of semi-invariant CD1d-restricted T cell receptors: the best of both worlds? Immunol Cell Biol. (2004) 82:285–94. doi: 10.1111/j.0818-9641.2004.01257.x 15186260

[7] Heczey A Liu D Tian G Courtney AN Wei J Marinova E . Invariant NKT cells with chimeric antigen receptor provide a novel platform for safe and effective cancer immunotherapy. Blood. (2014) 124:2824–33. doi: 10.1182/blood-2013-11-541235 25049283 PMC4215313

[8] Niedzielska M Chalmers A Popis MC Altman-Sharoni E Addis S Beulen R . CAR-iNKT cells: redefining the frontiers of cellular immunotherapy. Front Immunol. (2025) 16:1625426. doi: 10.3389/fimmu.2025.1625426 40718496 PMC12291068

[9] Wang X Bishop KA Hegde S Rodenkirch LA Pike JW Gumperz JE . Human invariant natural killer T cells acquire transient innate responsiveness via histone H4 acetylation induced by weak TCR stimulation. J Exp Med. (2012) 209:987–1000. doi: 10.1084/jem.20111024 22508835 PMC3348100

[10] Sharma A Lawry SM Klein BS Wang X Sherer NM Zumwalde NA . LFA-1 ligation by high-density ICAM-1 is sufficient to activate IFN-gamma release by innate T lymphocytes. J Immunol. (2018) 201:2452–61. doi: 10.5005/jp/books/12582_18 PMC6179935 30171164

[11] Pearce EL Poffenberger MC Chang CH Jones RG . Fueling immunity: insights into metabolism and lymphocyte function. Science. (2013) 342:1242454. doi: 10.1126/science.1242454 24115444 PMC4486656

[12] Geltink RIK Kyle RL Pearce EL . Unraveling the complex interplay between T cell metabolism and function. Annu Rev Immunol. (2018) 36:461–88. doi: 10.1146/annurev-immunol-042617-053019 29677474 PMC6323527

[13] Fox CJ Hammerman PS Thompson CB . Fuel feeds function: energy metabolism and the T-cell response. Nat Rev Immunol. (2005) 5:844–52. doi: 10.1038/nri1710 16239903

[14] MacIver NJ Michalek RD Rathmell JC . Metabolic regulation of T lymphocytes. Annu Rev Immunol. (2013) 31:259–83. doi: 10.1146/annurev-immunol-032712-095956 23298210 PMC3606674

[15] Rathmell JC Vander Heiden MG Harris MH Frauwirth KA Thompson CB . In the absence of extrinsic signals, nutrient utilization by lymphocytes is insufficient to maintain either cell size or viability. Mol Cell. (2000) 6:683–92. doi: 10.1016/s1097-2765(00)00066-6 11030347

[16] Peng M Yin N Chhangawala S Xu K Leslie CS Li MO . Aerobic glycolysis promotes T helper 1 cell differentiation through an epigenetic mechanism. Science. (2016) 354:481–4. doi: 10.1126/science.aaf6284 27708054 PMC5539971

[17] Chang CH Curtis JD Maggi LB Jr. Faubert B Villarino AV O'Sullivan D . Posttranscriptional control of T cell effector function by aerobic glycolysis. Cell. (2013) 153:1239–51. doi: 10.1016/j.cell.2013.05.016 23746840 PMC3804311

[18] Guo C Chen S Liu W Ma Y Li J Fisher PB . Immunometabolism: a new target for improving cancer immunotherapy. Adv Cancer Res. (2019) 143:195–253. doi: 10.1016/bs.acr.2019.03.004 31202359 PMC6822174

[19] Pavlova NN Thompson CB . The emerging hallmarks of cancer metabolism. Cell Metab. (2016) 23:27–47. doi: 10.1016/j.cmet.2015.12.006 26771115 PMC4715268

[20] Vander Heiden MG Cantley LC Thompson CB . Understanding the Warburg effect: the metabolic requirements of cell proliferation. Science. (2009) 324:1029–33. doi: 10.1126/science.1160809 19460998 PMC2849637

[21] Chang CH Qiu J O'Sullivan D Buck MD Noguchi T Curtis JD . Metabolic competition in the tumor microenvironment is a driver of cancer progression. Cell. (2015) 162:1229–41. doi: 10.1016/j.cell.2015.08.016 26321679 PMC4864363

[22] Lynch L Nowak M Varghese B Clark J Hogan AE Toxavidis V . Adipose tissue invariant NKT cells protect against diet-induced obesity and metabolic disorder through regulatory cytokine production. Immunity. (2012) 37:574–87. doi: 10.1016/j.immuni.2012.06.016 22981538 PMC4991771

[23] Kumar A Pyaram K Yarosz EL Hong H Lyssiotis CA Giri S . Enhanced oxidative phosphorylation in NKT cells is essential for their survival and function. Proc Natl Acad Sci USA. (2019) 116:7439–48. doi: 10.1073/pnas.1901376116 30910955 PMC6462103

[24] Kane H LaMarche NM Ni Scannail A Garza AE Koay HF Azad AI . Longitudinal analysis of invariant natural killer T cell activation reveals a cMAF-associated transcriptional state of NKT10 cells. Elife. (2022) 11. doi: 10.7554/elife.76586 36458691 PMC9831610

[25] Khurana P Burudpakdee C Grupp SA Beier UH Barrett DM Bassiri H . Distinct bioenergetic features of human invariant natural killer T cells enable retained functions in nutrient-deprived states. Front Immunol. (2021) 12:700374. doi: 10.3389/fimmu.2021.700374 34434191 PMC8380770

[26] Gumperz JE Miyake S Yamamura T Brenner MB . Functionally distinct subsets of CD1d-restricted natural killer T cells revealed by CD1d tetramer staining. J Exp Med. (2002) 195:625–36. doi: 10.1084/jem.20011786 11877485 PMC2193772

[27] Montoya CJ Pollard D Martinson J Kumari K Wasserfall C Mulder CB . Characterization of human invariant natural killer T subsets in health and disease using a novel invariant natural killer T cell-clonotypic monoclonal antibody, 6B11. Immunology. (2007) 122:1–14. doi: 10.1111/j.1365-2567.2007.02647.x 17662044 PMC2265989

[28] O'Reilly V Zeng SG Bricard G Atzberger A Hogan AE Jackson J . Distinct and overlapping effector functions of expanded human CD4+, CD8alpha+ and CD4-CD8alpha- invariant natural killer T cells. PLoS One. (2011) 6:e28648. doi: 10.1371/journal.pone.0028648 22174854 PMC3236218

[29] Baiu DC Sharma A Schehr JL Basu J Smith KA Ohashi M . Human CD4(+) iNKT cell adoptive immunotherapy induces anti-tumour responses against CD1d-negative EBV-driven B lymphoma. Immunology. (2024). doi: 10.1111/imm.13799 PMC11223969 38736328

[30] Baiu DC Smith KA Ferguson SA Schwartz NR Tripathy S Brand J . CD8+ T-cell antitumor immunity via human iNKT-DC conjugates. Cancer Immunol Res. (2026) 14:318–34. doi: 10.1158/2326-6066.cir-25-0454 41236542 PMC12716482

[31] Wen X Kim S Xiong R Li M Lawrenczyk A Huang X . A subset of CD8alphabeta+ invariant NKT cells in a humanized mouse model. J Immunol. (2015) 195:1459–69. doi: 10.4049/jimmunol.1500574 26157173 PMC4530047

[32] Arguello RJ Combes AJ Char R Gigan JP Baaziz AI Bousiquot E . SCENITH: a flow cytometry-based method to functionally profile energy metabolism with single-cell resolution. Cell Metab. (2020) 32:e7.1063–75. doi: 10.1016/j.cmet.2020.11.007 PMC8407169 33264598

[33] Burton EM Gewurz BE . Epstein-Barr virus oncoprotein-driven B cell metabolism remodeling. PLoS Pathog. (2022) 18:e1010254. doi: 10.1371/journal.ppat.1010254 36264852 PMC9584359

[34] Ma SD Xu X Plowshay J Ranheim EA Burlingham WJ Jensen JL . LMP1-deficient Epstein-Barr virus mutant requires T cells for lymphomagenesis. J Clin Invest. (2015) 125:304–15. doi: 10.1172/jci76357 25485679 PMC4382240

[35] Ma SD Xu X Jones R Delecluse HJ Zumwalde NA Sharma A . PD-1/CTLA-4 blockade inhibits Epstein-Barr virus-induced lymphoma growth in a cord blood humanized-mouse model. PLoS Pathog. (2016) 12:e1005642. doi: 10.1371/journal.ppat.1005642 27186886 PMC4871349

[36] Zumwalde NA Sharma A Xu X Ma S Schneider CL Romero-Masters JC . Adoptively transferred Vgamma9Vdelta2 T cells show potent antitumor effects in a preclinical B cell lymphomagenesis model. JCI Insight. (2017) 2. doi: 10.1172/jci.insight.93179 28679955 PMC5499361

[37] Oikonomakos NG Zographos SE Johnson LN Papageorgiou AC Acharya KR . The binding of 2-deoxy-D-glucose 6-phosphate to glycogen phosphorylase b: kinetic and crystallographic studies. J Mol Biol. (1995) 254:900–17. doi: 10.1006/jmbi.1995.0665 7500360

[38] Wang Z Guan D Wang S Chai LYA Xu S Lam KP . Glycolysis and oxidative phosphorylation play critical roles in natural killer cell receptor-mediated natural killer cell functions. Front Immunol. (2020) 11:202. doi: 10.3389/fimmu.2020.00202 32153568 PMC7045049

[39] Ma R Ji T Zhang H Dong W Chen X Xu P . A Pck1-directed glycogen metabolic program regulates formation and maintenance of memory CD8(+) T cells. Nat Cell Biol. (2018) 20:21–9. doi: 10.1038/s41556-017-0002-2 29230018

[40] Zhang H Liu J Yang Z Zeng L Wei K Zhu L . TCR activation directly stimulates PYGB-dependent glycogenolysis to fuel the early recall response in CD8(+) memory T cells. Mol Cell. (2022) 82:e6.3077–88. doi: 10.1016/j.molcel.2022.06.002 35738262

[41] Cassidy FC Kedia-Mehta N Bergin R Woodcock A Berisha A Bradley B . Glycogen-fuelled metabolism supports rapid mucosal-associated invariant T cell responses. Proc Natl Acad Sci USA. (2023) 120:e2300566120. doi: 10.1073/pnas.2300566120 37307453 PMC10288641

[42] Zhou Y Zhang C He L Kang Y Wang D Wang S . Glucose-1-phosphate promotes compartmentalization of glycogen with the pentose phosphate pathway in CD8(+) memory T cells. Mol Cell. (2025) 85:2535–49:e10. doi: 10.1016/j.molcel.2025.05.019 40499549

[43] Tahir SM Cheng O Shaulov A Koezuka Y Bubley GJ Wilson SB . Loss of IFN-gamma production by invariant NK T cells in advanced cancer. J Immunol. (2001) 167:4046–50. doi: 10.4049/jimmunol.167.7.4046 11564825

[44] Molling JW Kolgen W van der Vliet HJ Boomsma MF Kruizenga H Smorenburg CH . Peripheral blood IFN-gamma-secreting Valpha24+Vbeta11+ NKT cell numbers are decreased in cancer patients independent of tumor type or tumor load. Int J Cancer. (2005) 116:87–94. doi: 10.1002/ijc.20998 15756674

[45] Parekh VV Wilson MT Olivares-Villagomez D Singh AK Wu L Wang CR . Glycolipid antigen induces long-term natural killer T cell anergy in mice. J Clin Invest. (2005) 115:2572–83. doi: 10.1172/jci24762 16138194 PMC1193878

[46] Sag D Krause P Hedrick CC Kronenberg M Wingender G . IL-10-producing NKT10 cells are a distinct regulatory invariant NKT cell subset. J Clin Invest. (2014) 124:3725–40. doi: 10.1172/jci72308 25061873 PMC4151203

[47] Li YR Zhou Y Kim YJ Zhu Y Ma F Yu J . Development of allogeneic HSC-engineered iNKT cells for off-the-shelf cancer immunotherapy. Cell Rep Med. (2021) 2:100449. doi: 10.1016/j.xcrm.2021.100449 34841295 PMC8607011

[48] Simonetta F Lohmeyer JK Hirai T Maas-Bauer K Alvarez M Wenokur AS . Allogeneic CAR invariant natural killer T cells exert potent antitumor effects through host CD8 T-cell cross-priming. Clin Cancer Res. (2021) 27:6054–64. doi: 10.1158/1078-0432.ccr-21-1329 34376537 PMC8563377

[49] Delfanti G Dellabona P Casorati G Fedeli M . Adoptive immunotherapy with engineered iNKT cells to target cancer cells and the suppressive microenvironment. Front Med (Lausanne). (2022) 9:897750. doi: 10.3389/fmed.2022.897750 35615083 PMC9125179

[50] Look A Burns D Tews I Roghanian A Mansour S . Towards a better understanding of human iNKT cell subpopulations for improved clinical outcomes. Front Immunol. (2023) 14:1176724. doi: 10.3389/fimmu.2023.1176724 37153585 PMC10154573

[51] Wang Z Zhang G . CAR-iNKT cell therapy: mechanisms, advantages, and challenges. Curr Res Transl Med. (2024) 73:103488. doi: 10.1016/j.retram.2024.103488 39662251

