# Supplementary material for: Human CD4+ iNKT cells have highly oxidative metabolism and use glycogen reserves to sustain IFN-γ production in nutrient-limited environments
Source: Front Immunol. 2026 Aug 17;17:1894113. doi: 10.3389/fimmu.2026.1894113 (PMC13527001; doi:10.3389/fimmu.2026.1894113)
Supplement: Supplementary file 1 [file DataSheet1.pdf]

Supplementary Figures

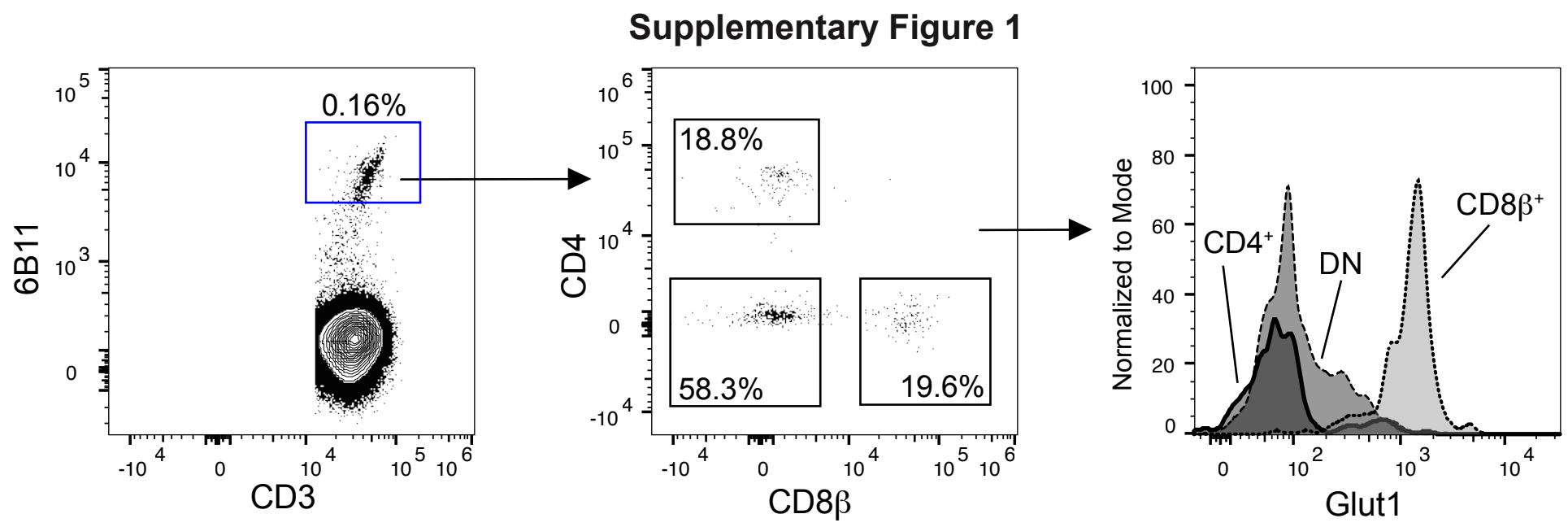

Figure S1. Flow cytometric analysis of cell surface Glut1 expression by CD4+, DN, and CD8β$^+$ iNKT cells in a PBMC sample that contains a high enough proportion of CD8β$^+$ iNKT cells for analysis.

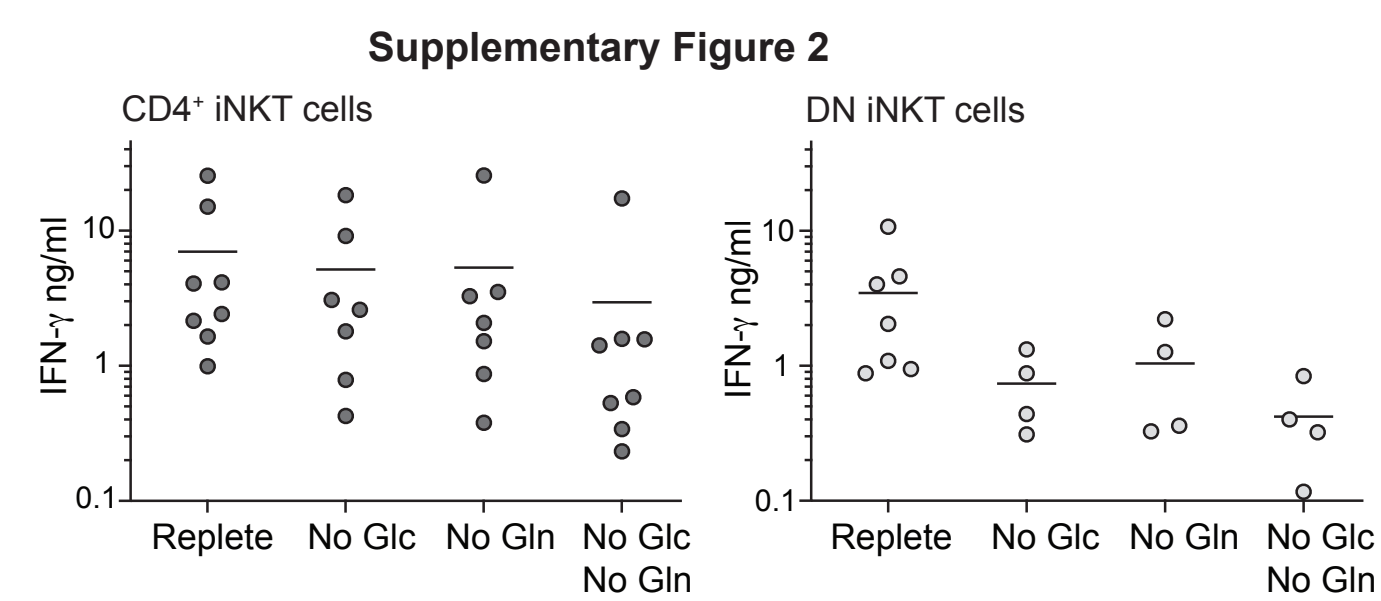

Figure S2. CD4$^+$ or DN iNKT cells were stimulated with anti-CD3/anti-CD28 antibody complex in medium lacking glucose, glutamine, or both. Each symbol shows result from an independent biological replicate. Plot shows concentration of IFN-γ detected in culture supernatants after 24h.

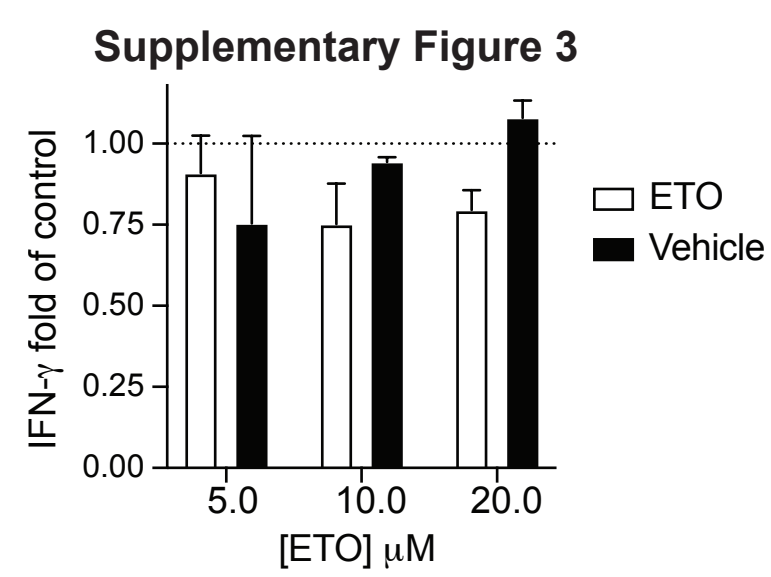

Figure S3. CD4$^+$ iNKT cells were stimulated with an anti-CD3/anti-CD28 antibody complex in the presence of the indicated concentrations of etomoxir (an inhibitor of fatty acid metabolism) or vehicle control. Plot shows amount of IFN-γ detected in culture supernatants as a fraction of that produced by untreated iNKT cells tested in parallel (dotted line). Bars show means±SD of 3 replicates.

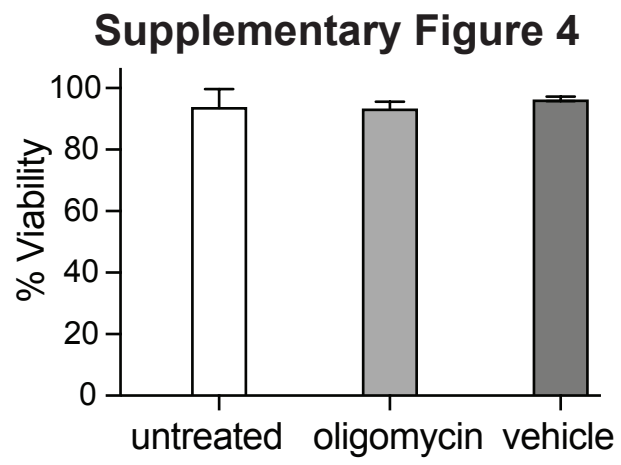

Figure S4. Viability of CD4$^+$ iNKT cells after 48h of oligomycin treatment compared to untreated or vehicle-treated iNKT cells cultured in parallel.

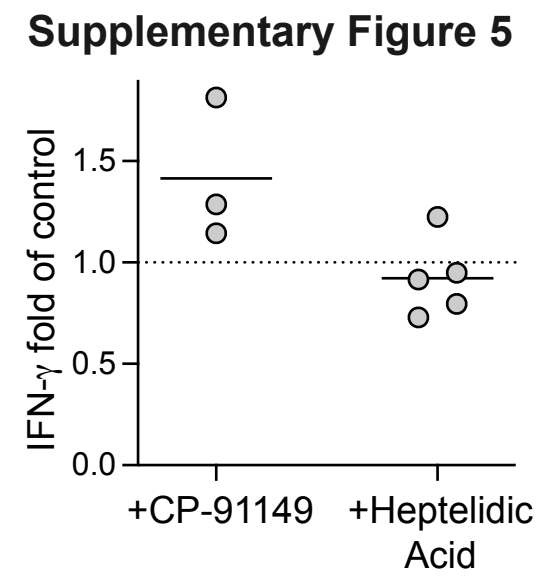

Figure S5. CD4$^+$ iNKT cells were stimulated with an anti-CD3/anti-CD28 antibody complex in nutrient-replete medium in the presence or absence of the CP-91149 or heptelidic acid inhibitors. Plot shows IFN-γ production relative to that by the same iNKT cells in the presence of vehicle.

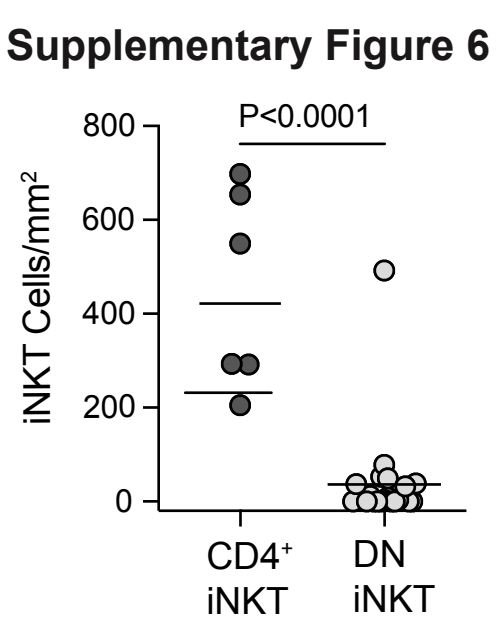

Figure S6. NSG mice were intraperitoneally injected with EBV-infected human cord blood mononuclear cells (CBMCs) resulting in the formation of EBV-driven human B cell lymphomas in the peritoneal cavity. Plot shows comparison of tumor infiltration by adoptively transferred CFSE-labeled CD4$^+$ vs. DN iNKT cells. Each symbol represents the number of labeled iNKT cells ennumerated in an independent 1 mm^2 area of lymphoma. P value determined by Mann-Whitney test.

Supplementary Figure 7

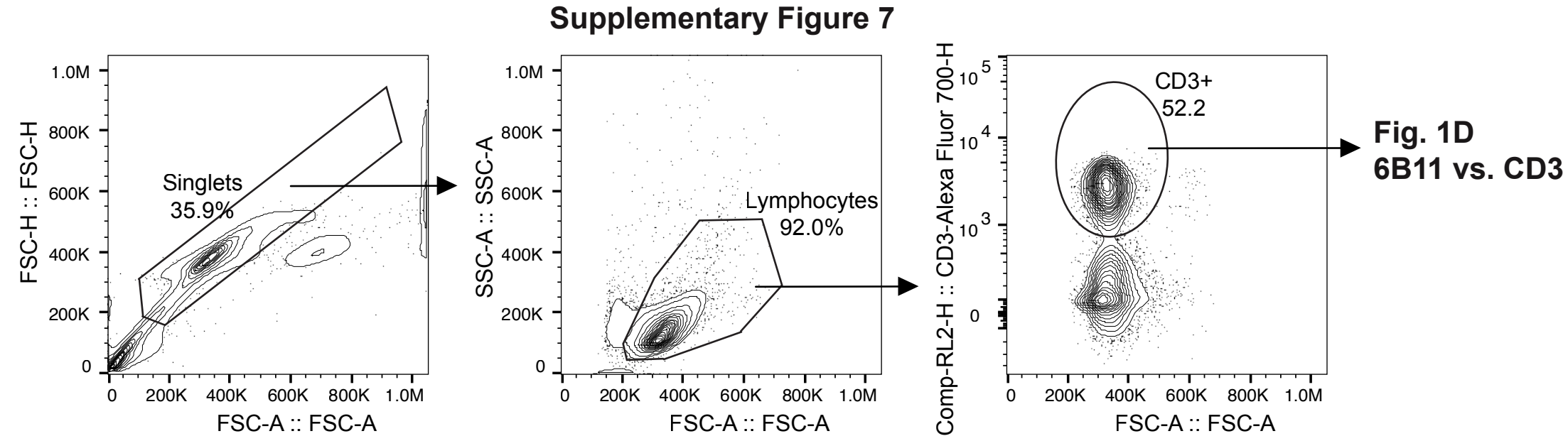

Figure S7. Flow cytometric staining of cell surface Glut1 expression by CD4^{+}, DN, and CD8β^{+} iNKT cells in a PBMC sample that contains a high enough proportion of CD8β^{+} iNKT cells for analysis.

Supplementary Figure 8

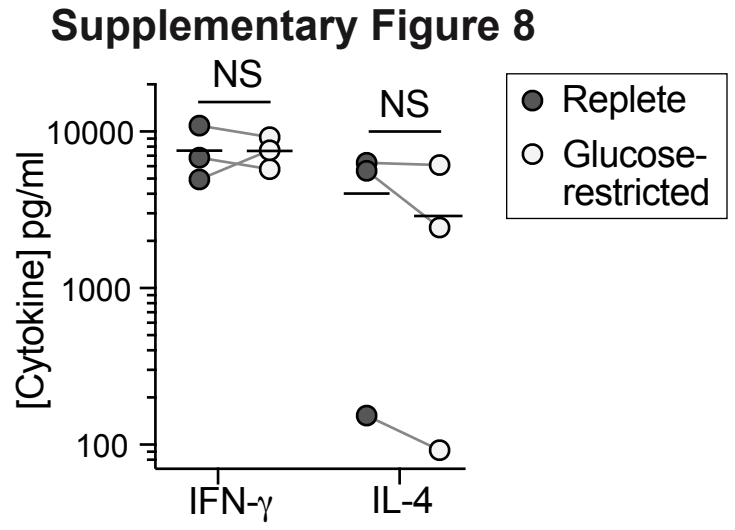

Figure S8. CD4^{+} iNKT cells were placed in nutrient-replete culture medium or in medium lacking glucose, and stimulated with an anti-CD3/anti-CD28 antibody complex for 24h. Paired symbols show mean amount of IFN-γ or IL-4 detected in the culture supernatant from independent experiments.
