# Supplementary material for: Human CD4+ iNKT cells have highly oxidative metabolism and use glycogen reserves to sustain IFN-γ production in nutrient-limited environments
Source: Front Immunol. 2026 Aug 17;17:1894113. doi: 10.3389/fimmu.2026.1894113 (PMC13527001; doi:10.3389/fimmu.2026.1894113)
Supplement: Supplementary file 2 [file DataSheet2.pdf]

Supplementary Table 1: Human iNKT cell lines

Donor 1	WINK1	CD4	Polyclonal
Donor 2	WINK2	CD4	Polyclonal
	WID5-CD4	CD4	Polyclonal
	WID5	DN	Polyclonal
	WINK2b.3	CD4	Clonal
	WINK2b.6	CD4	Clonal
Donor 3	WINK3	CD4	Polyclonal
	WINK3b.1	CD4	Clonal
	WINK3b.2	CD4	Clonal
	WINK3b.4	CD4	Clonal
	WINK3b.5	CD4	Clonal
	WINK3b.8	CD4	Clonal
	WINK3b.9	CD4	Clonal
	WINK3b.10	CD4	Clonal
Donor 4	WID4	CD4	Polyclonal
Donor 5	WINK4P1	CD4	Polyclonal
	WINK4P2	DN	Polyclonal
	WINK4C1.1	CD4	Clonal
	WINK4C2.1	DN	Clonal
Donor 6	WID3	CD4	Polyclonal
